# *FKBP5* polymorphisms induce differential glucocorticoid responsiveness in primary CNS cells – First insights from novel humanized mice

**DOI:** 10.1111/ejn.14999

**Published:** 2020-10-27

**Authors:** Verena Nold, Nadine Richter, Bastian Hengerer, Iris‐Tatjana Kolassa, Kelly Ann Allers

**Affiliations:** ^1^ Boehringer Ingelheim Pharma GmbH & Co KG CNSDR Ingelheim Germany; ^2^ Institute of Psychology & Education, Clinical & Biological Psychology Ulm University Ulm Germany

**Keywords:** astrocyte, CNS cell types, *Fkbp5*, glucocorticoid responsiveness, psychiatric disorders

## Abstract

The brain is a central hub for integration of internal and external conditions and, thus, a regulator of the stress response. Glucocorticoids are the essential communicators of this response. Aberrations in glucocorticoid signaling are a common symptom in patients with psychiatric disorders. The gene *FKBP5* encodes a chaperone protein that functionally inhibits glucocorticoid signaling and, thus, contributes to the regulation of stress. In the context of childhood trauma, differential expression of *FKBP5* has been found in psychiatric patients compared to controls. These variations in expression levels of *FKBP5* were reported to be associated with differences in stress responsiveness in human carriers of the single nucleotide polymorphism (SNP) rs1360780. Understanding the mechanisms underlying *FKBP5* polymorphism‐associated glucocorticoid responsiveness in the CNS will lead to a better understanding of stress regulation or associated pathology. To study these mechanisms, two novel humanized mouse lines were generated. The lines carried either the risk (A/T) allele or the resilient (C/G) allele of rs1360780. Primary cells from CNS (astrocytes, microglia, and neurons) were analyzed for their basal expression levels of *FKBP5* and their responsiveness to glucocorticoids. Differential expression of *FKBP5* was found for these cell types and negatively correlated with the cellular glucocorticoid responsiveness. Astrocytes revealed the strongest transcriptional response, followed by microglia and neurons. Furthermore, the risk allele (A/T) was associated with greater induction of *FKBP5* than the resilience allele. Novel *FKBP5*‐humanized mice display differential glucocorticoid responsiveness due to a single intronic SNP. The vulnerability to stress signaling in the shape of glucocorticoids in the brain correlated with *FKBP5* expression levels. The strong responsiveness of astrocytes to glucocorticoids implies astrocytes play a prominent role in the stress response, and in *FKBP5*‐related differences in glucocorticoid signaling. The novel humanized mouse lines will allow for further study of the role that *FKBP5* SNPs have in risk and resilience to stress pathology.

## Graphical abstract

Differential induction of FKBP5 as function of cellular identity and genotype regulates glucocorticoid responsiveness in primary CNS cell types.

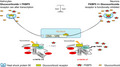

## 1 INTRODUCTION

Glucocorticoids are secreted following the diurnal rhythmicity of the hypothalamus‐pituitary‐adrenal (HPA) axis, and on demand during challenging situations that require allostatic adjustment. Glucocorticoids influence a plethora of physiological processes via transactivation or transrepression of gene expression. A high demand on the stress response system, such as with chronic exposure to stress, can lead to allostatic over‐load and stress‐related pathologies (Juster et al., 2010; McEwen, 2004; Nold et al., 2019). In stress‐associated disorders like post‐traumatic stress disorder (PTSD) or depression, dysregulation of the HPA axis is a common shared pathology, although the specifics of this dysregulation can range from hypo‐ to hyper‐sensitivity (Pariante, (2009); Perrin et al., 2019). As chronic stress has a direct impact on HPA‐axis function, it is considered to be an environmental risk factor for the development of many disorders (Chandola et al., 2006; Cohen et al., 2012; Lagraauw et al., 2015; Machado et al., 2014). The brain is a central hub for integration of psychological stress perception and consequent physiological stress responses. This can be demonstrated, for example, at the circuit level by combining neuroimaging with paradigms meant to induce stress (Rauch et al., 2006). Of course, these circuit‐level responses are not only purely driven by neuronal activity but also have a glial cell contribution. It is known that at the cellular level, either diurnal circulating or stress‐induced glucocorticoids impact neurons, microglia, and astrocytes to a different extent (Lucassen et al., 2014; Radley et al., 2015; Ressler & Smoller, 2016). Hence, the sensitivity to glucocorticoids, and regulators of such sensitivity, may be addressed at the cellular level. The chaperone FK506‐binding protein 51 (FKBP51), which is encoded by the *FKBP5* gene, is a potent negative regulator of glucocorticoid function. FKBP51 binds to heat‐shock protein 90 (Hsp90) complexes, and works within this complex to aid in folding or stabilizing a number of ‘client’ proteins (Lorenz et al., 2014). When the client protein is the glucocorticoid receptor, the receptor is expected to be in a low‐affinity state. Release of FKBP51 from this complex configuration is necessary for the glucocorticoid receptor to be active (Fries et al., 2017; Pirkl & Buchner, 2001; Scammell et al., 2003; Wochnik et al., 2005). As *FKBP5* is a target of glucocorticoid‐mediated transcription, activation of the glucocorticoid receptor with its ligand induces *FKBP5* and, subsequently, its protein FKBP51, again providing functional inhibition. Therefore, *FKBP5*/FKBP51 represents an ultra‐short negative feedback loop within the cell to terminate glucocorticoid signaling (Chun et al., 2011; Jaaskelainen et al., 2011; Yeo et al., 2017). Expression levels of *FKBP5* are, therefore, a critical setscrew for glucocorticoid sensitivity.

In the human population, there is a wide variation in perception of stressful challenges, and variation in physiological expression of stress. Single nucleotide polymorphisms (SNPs) within the *FKBP5* gene locus have been reported to impact stress responsivity, and risk or resilience to psychiatric disorders (Appel et al., 2011; Binder, 2009; Criado‐Marrero et al., 2019; Liebermann et al., 2017; Wilker et al., 2014). Differential expression levels of *FKBP5* have preclinically been shown to impact on stress‐coping behavior and the SNP rs1360780 was reported to profoundly influence stress coping, suggesting *FKBP5* genotypes as one source of human variation (Ising et al., 2008; Touma et al., 2011). In combination with early‐life adversity, demethylation of *FKBP5* has been reported (Hohne et al., 2014; Klengel et al., 2013), which could lead to a higher expression of *FKBP5* rendering the affected individual more prone to glucocorticoid resistance and psychiatric disorders (Binder et al., 2008; Zimmermann et al., 2011). The ability of the A/T or C/G rs1360780 allele to impart differential stress responses could contribute to the wide variation in stress responses observed in the human population.

These SNPs do not exist in laboratory rodents. Hence, in order to study the effect of these human gene variants preclinically, two novel humanized mouse lines carrying the risk‐associated rs1360780‐A/T or the resiliency‐associated rs1360780‐C/G allele of the human *FKBP5* were developed at Taconic Biosciences. Hereafter, these lines are referred to as risk (A/T) and resilience (C/G), respectively. There were three goals in these first studies: (1) establish that the two mouse lines, carrying only a single nucleotide difference, responded to glucocorticoid stimulation, therefore, validating both the functional polymorphism and intact glucocorticoid signaling following humanization; (2) determine if, in primary cell culture, differential responses between risk (A/T) and resilience (C/G) could be observed; and (3) assess relative expression across cell types and association with glucocorticoid responsiveness. In these novel strains, we investigated whether the human risk or resiliency version of *FKBP5* would influence acute glucocorticoid responsiveness of primary astrocytes, microglia, and neurons. Based on the inhibitory capacity of *FKBP5* on glucocorticoid signaling, we hypothesized that high basal expression level of *FKBP5* would be associated with a decreased responsiveness to glucocorticoids and that the risk (A/T) allele of *FKBP5* should show a higher reactivity to glucocorticoids than the resiliency allele. A more far reaching goal is for these mice to serve as a unique tool to further study the influence of the human *FKBP5* gene variants on the risk and resilience to stress‐related disorders.

## 2 MATERIALS AND METHODS

### 2.1 Generation of FKBP5 – Transgenic mice

Taconic Biosciences was commissioned to generate two novel transgenic mouse models carrying either the cytosine (C)/guanidine (G) variant at position 3622 in the human FKBP5 gene (Ensembl gene ID: ENSMUSG00000024222; NCBI gene ID: 14229) or the risk‐associated adenine (A)/thymidine (T) version of rs1360780 (Insertion gene identifier: ENSG00000096060 [Ensembl gene ID]; 2289 [NCBI gene ID]). This process was performed under the scientific guidance of Dr. Elisabeth Binder from the Max Planck Institute for Psychiatry in Munich. The targeting strategy for the constitutively humanized FKBP5 gene was based on the NCBI transcript NM_010220_4 (Ensembl transcript ENSMUST00000079413, Fkbp5_001) for the mouse and the transcript NM_001145775_2 (ENSMUST00000536438, Fkbp5_201) for the human gene. Using BAC clones from the mouse C57BL/6J RPCI‐23 and human RPCI‐11 BAC and/or CalTechD libraries, a targeting vector was generated. The mouse genomic region between exon 2, containing the translation initiation codon, and exon 11, containing the termination codon, has been replaced with the human counterpart containing a neomycin resistance (NeoR, flanked by FRT, exon 3) and a puromycin resistance (PuroR, flanked by F3, exon 11) for positive selection of clones. A schematic map of the targeting vector (Figure SA1) and the sequence (Supplementary Material Text 1) is provided. The linearized DNA targeting vector was transfected via electroporation into the Taconic Biosciences C57BL/6N Tac ES cell line, incorporated via homologous recombination, and recombinant clones were isolated based on double‐positive (NeoR and PuroR) and negative (Thymidine kinase) selections. Single integration and homologous recombination at the 5′ and 3′ side were assessed via digestion with restriction enzymes (BauI, MfeI, KpnI, SpeI, EcoRI, BmtI, ScaI, EcoRV, and PacI) and southern blotting as well as PCR‐based sequencing of the 5′ and 3′ junction between human and murine regions. The selected heterozygote‐targeted ES cells were transiently transfected with the circular vector pCAG‐Flpe‐pA (3465) containing a Flp recombinase targeting the F3, F5, or FRT sites as well as the vectors pCAG‐Cre for loxP sites and phiC31 for attB/attP sites, via nucleofection for in vitro removal of the selection markers. Recovery of the humanized allele was confirmed using PCRs and tested for sensitivity to the respective antibiotics. The selected ES cells containing the humanized allele were transferred into blastocysts and the resulting chimeras were used for breeding with C57BL/6NTac mice and founding the novel mouse strain C57BL/6NTac‐Fkbp5tm4570 (FKBP5) Tac, carrying the risk‐associated rs1360780‐A/T or the mouse‐line C57BL/6NTac‐Fkbp5tm4571 (FKBP5) Tac carrying the resiliency version, respectively. Breeding of the homozygote humanized mice and their wild types was performed in house. The ownership of these animals was transferred to Taconic to make them publicly available.

### 2.2 Primary cell cultures

Protocols were validated in house for optimal yield and development of cell type‐specific features and experiments were performed under the allowance of the regional council for animal welfare (Regierungspräsidium Tübingen, Baden‐Württemberg, Germany).

To obtain single cell suspensions for neuronal cultures, cortex and hippocampus of embryos at E16.5 were dissected, enzymatically digested, and mechanically dissociated. If viability was above 90%, cells were seeded into PDL‐Laminin–coated plates in a density of 100,000 cells per 24‐well and incubated at 37°C and 5% CO_2_. Half exchanges of the serum‐free culture medium (5 ml GlutaMax, 10 ml SM1‐Supplement [#05711; Stemcell Technologies, Köln, Germany], 5 ml HEPES 1 M [#83264‐100Ml‐F; Sigma Aldrich, Taufkirchen, Germany], and 500 ml Neurobasal [#12348017; Invitrogen]) were performed in intervals of 3–4 days.

Cell suspensions for glial cultures were obtained by isolating cells from the cortices of neonates via enzymatic digestion with DNase (#LS002139; Worthington, NJ, USA) and 2.5% trypsin (#15090046; Invitrogen) followed by mechanical homogenization in flask medium (10% FCS, 5 ml Penicillin/Streptomycin, 5 ml HEPES, and 500 ml advanced DMEM [#12491015; Invitrogen]). Cells were cultured in precoated flask (75 cm^2^ flasks, Poly‐L‐Ornithin Hydrobromid [MW: ≤30,000–70,000 Dalton, #P3655; Sigma]) at 37°C with 5% CO_2_. Every 3–4 days, the flasks were washed with PBS (#14040174; Invitrogen) and refilled with flask medium. Microglia were harvested in 3–4 days and plated at a density of 150,000 cells per 24‐well of uncoated PRIMARIA plates (#353847; Corning, Germany).

For plating of astrocytes, remaining microglia were shaken off, flasks were washed, and the astrocyte layer was detached using 0.05% trypsin‐EDTA solution (#25300054; Invitrogen). Astrocytes were suspended in 50‐ml advanced DMEM containing 10% FCS to stop trypsination per flask and 1 ml of this suspension was used per 24‐well of a PRIMARIA plate. On the next day, a full medium exchange was performed. On post‐plating day (PPD) 8, a confluent astrocyte layer was obtained and cells were exposed to AraC medium (#251010; Cytosine Arabinoside, Sigma, 8 μM) for 4 days. On PPD11, the medium was exchanged to LME medium (L‐leucine methyl esters, #L1002; Sigma, 50 mM) for 1 hr and astrocytes were subsequently washed three times with medium. On PPD14, the medium was exchanged to serum‐free medium and the assay was performed the next day.

### 2.3 Stimulation with glucocorticoids

Stimulation was performed between 08:00 a.m. to 10:00 a.m. with neuronal cultures being stimulated on day in vitro 12, while microglia were stimulated 1 day and astrocytes 15 days after plating, respectively. Stocks of glucocorticoids in dimethyl sulfoxide (DMSO) were freshly diluted 1:200 in the respective warmed culture medium and cells were stimulated by replacing 0.5 ml of the medium in the well with the obtained agonist solutions so that final concentrations of 0.8, 4, 20, or 100 nM for dexamethasone and corticosterone or 5.6, 28, 140, and 700 nM for prednisolone were obtained. To control for manipulation of vehicle effects, a half medium exchange was performed or cells were treated with medium containing 0.005% DMSO, respectively. To investigate transcriptional responses to an acute challenge, after 4 hr of incubation cells were lysed in 250 µl RLT buffer (#79216; Qiagen, Hilden, Germany) containing 1% beta‐mercapto‐ethanol (#M3148‐100Ml; Sigma), and frozen at −20°C prior to RNA isolation. No sample had to be excluded from analyses due to diminished viability after stimulation, as assessed by visual inspection and occasional lactate‐dehydrogenase–based viability assays.

### 2.4 Reverse transcription‐quantitative polymerase chain reaction (RT‐qPCR)

RNA was isolated using RNeasy Plus kit (#74192; Qiagen) following the manufacturer's recommendations. Prior to reverse transcription of the RNA, integrity was confirmed to be above a RNA integrity number of 8 on occasion using the Fragment analyzer (Thermo Fisher Scientific, Langenselbold, Germany) and the obtained yield was determined spectrophotometrically (QIAxpert, Qiagen) to allow normalization of the input RNA concentration to 500 ng. Reverse transcription of the total mRNA to complementary DNA was performed using the high‐capacity cDNA kit (#4368813; Qiagen). All TaqMan gene expression assays were labeled with FAM (#4352042; Thermo Fisher) and used in conjunction with the fast universal PCR Master Mix (#4351368; Thermo Fisher). The used primers were as follows: succinate dehydrogenase complex subunit A (Sdha, Mm01352366_m1), human FKBP5 (Hs01561006_m1), murine Fkbp5 (Mm00487403_m1), FK506 binding protein 4 (Fkbp4, Mm00487391_m1), glucocorticoid receptor (Nr3c1, Mm00433832_m1), mineralocorticoid receptor (Nr3c2, Mm01241596_m1), glucocorticoid‐induced leucine zipper (Tsc22d3, Mm01306210_g1), nuclear factor‐κ‐B‐inhibitor α (NFkBia, Mm00477798_m1), period 1 (Per1, Mm00501813_m1), sestrin 1 (Sesn1, Mm01185732_m1), and serum and GC‐regulated kinase 1 (Sgk1, Mm00441380_m1). Samples were analyzed in technical triplicates on a QuantStudio 6 (Thermo Fisher). All gene expression levels were normalized relative to the cycle thresholds measured for Sdha and relative to DMSO‐treated cells within the same cell type, genotype, and mouse strain for stimulation experiments.

### 2.5 Statistics

Technical replicates of stimulations, RNA isolations, cDNA, and TaqMan were summarized per sample so that each individual data point shown and used for analysis represent one individual biological sample. Thus, the number of data points represents the replicates per group. All data visualization and analysis were performed using R version 3.6.1. Distribution of data points and model residuals were tested for departure from normality and homogeneity of variances using a Shapiro‐Wilk test, visual inspection, as well as the Bartlett's test or Levene's test, respectively. One‐way ANOVA of type II for baseline values of Fkbp5 in wild‐type cells was used to compare between untreated (native) and DMSO‐treated cells. A Kruskal–Wallis ranked ANOVA was used for comparing the dCT data of the glucocorticoid receptor expression due to not normally distributed residuals. A two‐way ANOVA of type I was used for the (ranked) fold‐change data with cell type and glucocorticoid dose as model factors. In case of significant findings in the ANOVA, the Tukey Honest Significant Difference test or Wilcoxon test was used post hoc to determine which groups differed and to adjust *p*‐values for multiple comparisons. The contrasts of interest were defined a priori. Rank‐based tests were applied when required. All tests were performed in a two‐sided manner.

## 3 RESULTS

### 3.1 Cell type‐specific expression of *Fkbp5* and *Nr3c1* in the CNS

**FIGURE 1:**
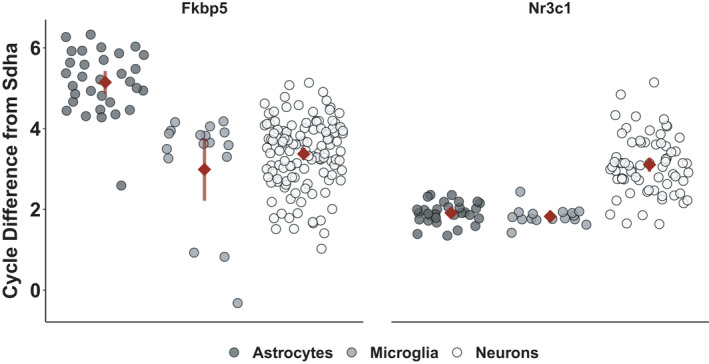
*Sdha*‐normalized mRNA expression levels of *Fkbp5* (left panel) and *Nr3c1* (right panel) of primary murine wild‐type astrocytes (dark grey, left), microglia (grey, middle), and neurons (ecru, right). Individual data points are shown alongside with their mean ± 95% CI (red). High values in the PCR cycles needed to reach the set threshold represent low amounts of the targeted mRNA and, hence, a low expression of the gene while low cycle numbers indicate higher expression

In primary murine wild‐type (WT) astrocytes, microglia, and neurons, the basal mRNA expression of the glucocorticoid receptor (*Nr3c1*) and its functional inhibitor FKBP51 (*Fkbp5*) was examined and is visualized as qPCR cycle number difference from the housekeeper *Sdha* (Figure 1). A one‐way ANOVA revealed that cell types differed in the expression of *Fkbp5* (*F*(3, 168) = 33.54; *p* < 0.0001) and a Tukey Honest Significance *Post Hoc* Test showed that astrocytes had the lowest expression of the inhibitory chaperone *Fkbp5* (5.14 ± 0.78) compared to microglia (2.99 ± 1.56, *p* < 0.0001) and neurons (3.37 ± 0.85, *p* < 0.0001). *Nr3c1* was also differentially expressed between the cell types (Kruskal–Wallis rank‐sum test χ^2^(3) = 64.07, *p* < 0.0001) with the highest expression in both glial cell types (astrocytes = 1.91 ± 0.26, microglia = 1.83 ± 0.22) compared to neurons (3.11 ± 0.72, *p* < 0.0001).

### 3.2 Induction of glucocorticoid response element containing genes differs across cell types

To determine responsiveness to glucocorticoids, the different cell types were stimulated with increasing doses of the synthetic glucocorticoid dexamethasone. In response, prominent fold changes in the expression of glucocorticoid‐responsive genes were observed (Table 1). A ranked ANOVA revealed an interaction of cell type and dose, *F*(8, 309) = 95.9, *p* < 0.0001, on the induction of *Fkbp5*. Subsequent analysis indicated that astrocytes (21.01 ± 10.52) differed from microglia (7.81 ± 8.09) and neurons (2.13 ± 1.76). Furthermore, all dexamethasone doses (10.85 ± 11.11) were different from vehicle (1.25 ± 1.28). For the glucocorticoid receptor, the ANOVA of ranks suggested differences between cell types, *F*(2, 237) = 37.2, *p* < 0.00001, and *post hoc* testing revealed astrocytes (0.72 ± 0.20) to have a reduced expression of *Nr3c1* after stimulation. For *Tsc22d3*, the ANOVA of ranks suggested a highly significant interaction of cell type and dose, *F*(8, 237) = 38.0, *p* < 0.0001, which was attributable *post hoc* to both glial cell types differing from neurons (2.73 ± 2.77). Astrocytes (16.92 ± 7.95) were more responsive than microglia (9.90 ± 10.31), and stimulated (11.09 ± 9.08) were different from vehicle‐treated (1.33 ± 0.96) cells. Fold changes of *Per1* showed a significant interaction of cell types and dexamethasone dose, *F*(8, 238) = 21.9, *p* < 0.0001, in the ANOVA of ranks that originated from stronger responses in astrocytes (4.42 ± 1.80) than in microglia (2.19 ± 1.36) and neurons (1.18 ± 0.54). The difference between vehicle‐treated (1.11 ± 0.50) and dexamethasone‐treated (5.07 ± 1.08) astrocytes was also demonstrated. Ranked fold changes of *Nfκbia* were found to vary between cell types and dexamethasone stimulation, *F*(8, 238) = 12.5, *p* < 0.0001, which was attributed to differences between astrocytes (3.12 ± 1.25) and microglia (2.03 ± 1.53) and differences between all glial cells and neurons (1.3 ± 0.47). Differences were also demonstrated between stimulated (2.32 ± 1.29) and vehicle‐treated (1.11 ± 0.73) cells. For the ranked fold changes of *Sgk1*, the same interaction was observed, *F*(8, 238) = 9.67, *p* < 0.00001, and confirmed *post hoc* to be based on astrocytic responsiveness to dexamethasone (3.50 ± 1.06) compared to vehicle‐treated astrocytes (1.04 ± 0.30).

**TABLE 1:** Dexamethasone‐induced fold changes of glucocorticoid response element containing genes in primary cell types of the central nervous system

Cell type	Astrocytes					Microglia					Neurons				
Dexamethasone (nM)	0	0.8	4	20	100	0	0.8	4	20	100	0	0.8	4	20	100
*Fkbp5* 304 *df*															
*N*	15	18	19	20	18	9	5	7	7	6	54	12	43	51	40
Mean	1.14	27.35	24.36	23.90	24.49	2.04	7.31	7.86	10.34	13.86	1.15	2.14	1.84	2.81	2.89
*SD*	0.84	5.95	6.86	6.11	5.04	3.21	4.14	5.28	9.45	12.16	0.71	0.62	1.44	2.36	1.73
LCL	−1.83	24.64	21.72	21.32	21.77	−1.80	2.16	3.51	5.98	9.16	−0.42	−1.19	0.09	1.20	1.07
UCL	4.12	30.07	27.00	26.47	27.20	5.88	12.46	12.22	14.69	18.57	2.71	5.46	3.60	4.43	4.71
*Nr3c1* 237 *df*															
*N*	14	18	18	19	18	9	5	7	7	7	33	12	27	31	27
Mean	1.01	0.70	0.69	0.67	0.62	1.02	0.83	0.91	0.90	0.85	1.39	1.16	1.41	1.43	1.16
*SD*	0.17	0.18	0.13	0.17	0.10	0.18	0.08	0.31	0.29	0.22	0.75	0.24	0.82	0.80	0.74
LCL	0.57	0.31	0.31	0.29	0.23	0.47	0.10	0.29	0.28	0.23	1.10	0.69	1.10	1.13	0.84
UCL	1.45	1.08	1.08	1.04	1.01	1.56	1.57	1.53	1.52	1.47	1.67	1.64	1.73	1.72	1.48
*Nfkbia* 238 *df*															
*N*	14	18	18	19	18	9	5	7	7	7	34	12	27	31	27
Mean	1.01	3.64	3.58	3.54	3.33	1.47	1.75	2.19	2.35	2.44	1.06	1.46	1.24	1.41	1.44
*SD*	0.14	0.87	0.98	0.92	0.94	1.73	0.51	1.45	1.74	1.77	0.36	0.46	0.47	0.48	0.51
LCL	0.36	3.07	3.01	2.98	2.76	0.66	0.66	1.27	1.44	1.53	0.64	0.76	0.78	0.97	0.98
UCL	1.66	4.21	4.15	4.09	3.90	2.28	2.83	3.11	3.27	3.36	1.48	2.16	1.71	1.84	1.91
*Per1* 238 *df*															
*N*	14	18	18	19	18	9	5	7	7	7	34	12	27	31	27
Mean	1.04	5.29	5.38	4.87	4.74	1.24	2.49	2.56	2.55	2.45	1.10	1.30	1.13	1.14	1.33
*SD*	0.3	1.13	0.94	1.17	1.08	0.81	1.03	1.22	1.90	1.41	0.47	0.43	0.54	0.48	0.70
LCL	0.37	4.69	4.79	4.30	4.15	0.40	1.37	1.61	1.60	1.50	0.67	0.57	0.65	0.69	0.84
UCL	1.71	5.88	5.97	5.45	5.33	2.08	3.61	3.51	3.50	3.40	1.53	2.02	1.62	1.59	1.81
*Sgk1* 238 *df*															
*N*	14	18	18	19	18	9	5	7	7	7	34	12	27	31	27
Mean	1.04	3.41	3.43	3.24	3.93	1.24	2.17	1.98	2.31	2.69	1.13	1.08	1.36	1.34	1.46
*SD*	0.3	0.92	0.88	0.85	1.61	1.01	1.19	1.36	1.95	2.46	0.61	0.64	0.89	0.73	0.86
LCL	0.24	2.70	2.73	2.55	3.22	0.25	0.84	0.86	1.18	1.57	0.62	0.21	0.78	0.81	0.88
UCL	1.84	4.11	4.14	3.92	4.63	2.24	3.51	3.11	3.44	3.82	1.64	1.94	1.93	1.88	2.03
*Tsc22d3* 237 *df*															
*N*	14	18	18	19	18	9	5	7	7	7	33	12	27	31	27
Mean	1.04	20.55	20.06	20.36	18.87	1.06	20.75	11.25	10.37	11.71	1.52	4.52	2.70	3.56	2.51
*SD*	0.35	3.68	3.10	4.75	4.82	0.32	10.43	9.90	10.29	10.20	1.18	1.49	2.79	4.03	2.19
LCL	−2.35	17.56	17.07	17.45	15.88	−3.17	15.08	6.45	5.57	6.91	−0.69	0.86	0.26	1.28	0.07
UCL	4.44	23.54	23.05	23.27	21.86	5.29	26.43	16.04	15.17	16.51	3.73	8.19	5.14	5.84	4.95

Abbreviations: nM, nano‐molar; *N*, number of observations; *SD*, standard deviation; *df*, degrees of freedom; LCL, lower 95% confidence level; ULC, upper 95% confidence level.

### 3.3 Expression levels of *Fkbp5* correlate with cellular glucocorticoid responsiveness

Correlation analyses of basal expression levels of *Fkbp5* and *Nr3c1* with induction of glucocorticoid response element containing genes indicated a strong relation (Table 2). As measure of glucocorticoid responsiveness, the cycle difference from housekeeper assessed after exposure to 100nM dexamethasone was used. The cycle difference after stimulation negatively correlated with the basal expression of *Fkbp5* for the genes *Nfkbia* (β = −0.46, *p* = 0.006), *Tsc22d3* (β = −0.42, *p* = 0.01), and *Sgk1* (β = −0.34, *p* = 0.05) and was marginally significant for *Nr3c1*, *Sesn1*, and *Fkbp5*. Including the basal levels of *Nr3c1* resulted in significant correlations of the differences from *Fkbp5* to *Nr3c1* with all tested glucocorticoid‐responsive genes. The difference of *Fkbp5* to *Nr3c1* expression levels at baseline strongly correlated with cell type (ρ = 0.8, *p* < 0.00001).

**TABLE 2:** Correlation of glucocorticoid response element containing gene induction after stimulation with 100 nM dexamethasone to basal *Fkbp5* and *Nr3c1* expression

Gene	Basal *Fkbp5*					Basal difference *Fkbp5‐Nr3c1*				
	Method	S/*t*	*df*	*p*‐value	β	Method	S/*t*	*df*	*p*‐value	β
*Fkbp5*	Spearman	9,434		0.06	−0.32	Spearman	11,000		0.001	−0.54
*Nfkbia*	Spearman	10,424		0.006	−0.46	Spearman	12,486		<0.00001	−0.75
*Nr3c1*	Pearson	−1.99	33	0.06	−0.33	Pearson	−5.96	33	<0.00001	−0.72
*Sesn1*	Pearson	−1.93	33	0.06	−0.32	Pearson	−6.04	33	<0.00001	−0.72
*Sgk1*	Pearson	−2.09	33	0.05	−0.34	Pearson	−3.44	33	0.002	−0.51
*Tsc22d3*	Spearman	10,122		0.01	−0.42	Spearman	11,114		0.0006	−0.56

Abbreviations: nM, nano‐molar; S, Spearman's rank correlation statistic sum of squared rank differences; *t*, Pearson's statistic *t*‐value; *df*, degrees of freedom; β, correlation estimate.

### 3.4 FKBP5 is expressed in CNS cells of humanized mice

In primary astrocytes, microglia and neurons derived from the *FKBP5*‐humanized mouse lines carrying either the risk (A/T) allele or the resiliency (C/G) allele the, same cell type‐specific expression differences of *FKBP5*, as observed for WT cells, were found (Figure SA2). An ANOVA of ranks suggested a significant difference in basal *FKBP5* expression, *F*(2, 101) = 33.80, *p* < 0.0001, and a trend of the transgenic strains expressing less *FKBP5* than wild type (*F*(1, 101) = 3.36, *p* = 0.07). The difference between cell types was *post hoc* found to originate from astrocytes (5.10 ± 1.35) differing from microglia (3.73 ± 0.62) and neurons (3.43 ± 0.60). To confirm that the humanized FKBP51 protein is expressed in these mice, Western Blot analyses were performed. An exemplary blot of prefrontal cortex, amygdala, and hippocampus homogenate from a humanized mouse and a wild‐type mouse 24 hr after stimulation with 0.1 mg/kg subcutaneous dexamethasone is provided (Figure SA3) together with a description of the method.

### 3.5 Differential responsiveness of human FKBP5 rs1360780 variants

**FIGURE 2:**
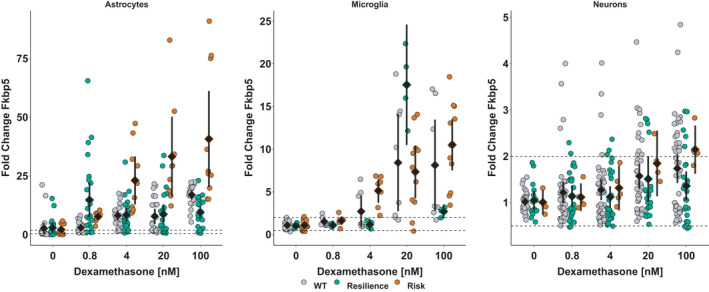
Dose‐responsive increase in *FKBP5* expression in primary murine astrocytes (left), microglia (middle), and neurons (right) after 4 hr stimulation with dexamethasone relative to expression of the housekeeper *Sdha* and *FKBP5* expression in DMSO‐only treated cells. Cells carrying the risk version of the human FKBP5 are depicted in orange while cells carrying the rs1360780‐C/G are depicted in green and cells derived from wild‐type mice are visualized in grey. Data points are shown alongside with their mean ± 95% CI. Dashed lines at 0.5 and 2 delimit the area of technically unclear change, which is based on the variability in *Sdha* expression levels

The responsiveness of these primary cell cultures to glucocorticoids was investigated by measuring the fold change of *FKBP5* expression after stimulation with dexamethasone (Figure 2). In astrocytes and microglia, a dose‐responsive increase in *FKBP5* mRNA expression was detected, which was stronger in cells carrying the risk (A/T) than the resilience (C/G) allele or the murine *Fkbp5*. In the ANOVA of ranks, an interaction between strains and dose was found in astrocytes, *F*(8, 260) = 14.1, *p* < 0.0001. *Post hoc* testing showed this was attributable to a significant difference between the risk (A/T) allele carrying astrocytes that were treated with 100 nM or 20 nM compared to wild‐type or resilience (C/G) allele carrying astrocytes. With respect to dose, only the risk (A/T) allele carrying astrocytes showed a dose‐responsive effect to dexamethasone (55.3 ± 28.6 at 100 nM versus 1.2 ± 0.7 in vehicle‐treated astrocytes). In resilience (C/G) allele carrying astrocytes or wild types, a dose‐responsive induction was visible but not of statistical significance and both strains did not differ from each other. In microglia, the interaction of strain and dexamethasone was significant (*F*(8, 123) = 7.0, *p* < 0.0001). This was due to differences in microglia carrying the risk (A/T) allele between vehicle‐treated (1.1 ± 0.5) and stimulated cells (100 nM dexamethasone: 10.5 ± 4.7; 20 nM dexamethasone: 7.3 ± 4.6). For neurons, no significant changes were observed.

### 3.6 Human SNP rs1360780 modifies cellular glucocorticoid responsiveness

**FIGURE 3:**
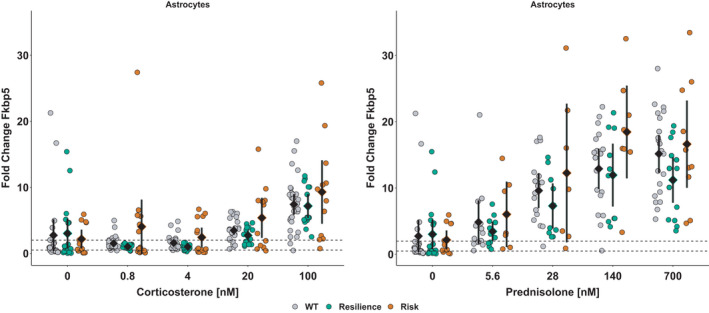
Astrocytes respond to a 4 hr stimulation with corticosterone (left) and prednisolone (right) in a dose‐responsive manner. Cells derived from humanized mice carrying rs1360780‐A/T (orange) trend to have higher fold changes in *FKBP5* mRNA expression than astrocytes carrying the resilience version with the C/G‐allele (green) or wild‐type astrocytes (grey). Data points are visualized alongside with their mean ± 95% CI. Dashed lines at 0.5 and 2 delimit the area of technically irrelevant change, as determined by variability of *Sdha* expression, as this gene was used as a house keeper to normalize for potential variation in utilized RNA concentrations

To rule out dexamethasone‐specific effects, the cellular responsiveness to other glucocorticoids was assessed. Stimulation with corticosterone or prednisolone resulted in a dose‐responsive increase in *FKBP5* expression in astrocytes with risk (A/T) allele carrying cells trending to respond more than resiliency (C/G) allele carriers (Figure 3). The ANOVA of ranks suggested a significant dose effect in astrocytes, *F*(4, 252) = 53.8, *p* < 0.0001, which was caused by differences between astrocytes stimulated with 100 nM corticosterone (7.8 ± 4.8) and astrocytes treated with lower amounts (20 nM: 3.7 ± 2.7; 4 nM: 1.7 ± 1.6; 0.8 nM: 2.0 ± 3.8) or vehicle (2.7 ± 4.5). Furthermore, the effect of strain was significant, *F*(2, 252) = 3.4, *p* < 0.04, originating from differences between wild types (3.4 ± 3.8) and risk (A/T) allele carriers (4.6 ± 5.8; *t*(690) = −3.3, *p* < 0.04). In microglia, the ANOVA of ranks suggested a significant interaction of corticosterone and strain, *F*(8, 110) = 13.0, *p* < 0.0001, based on differences between microglia treated with 100 nM corticosterone (8.6 ± 5.4) and lower doses (20 nM: 4.0 ± 2.0; 4 and 0.8 nM: 1.6 ± 0.4) or vehicle (1.1 ± 0.4) that varied between strains. In neurons, no changes bigger than technical resolution were found. For prednisolone, the ANOVA of ranks suggested significant influence of strain, *F*(2, 181) = 5.2, *p* < 0.007, with risk (A/T) allele carrying astrocytes showing significant responses to prednisolone at concentration of 700 nM (16.6 ± 9.2) and 140 nM (18.5 ± 8.4) compared to vehicle‐treated (2.2 ± 2.2) but not in resilience (C/G) allele carrying astrocytes.

### 3.7 Functional induction of other glucocorticoid response element harboring genes

**FIGURE 4:**
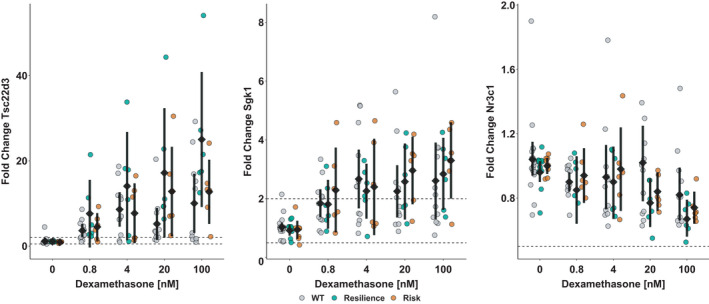
Astrocytes respond to a 4 hr stimulation with dexamethasone in a dose‐responsive manner with changes in gene expression of *Tsc22d3* (left), *Sgk1* (middle), and *Nr3c1* (right). Cells derived from humanized mice carrying rs1360780‐C/G (green) have higher fold changes in *Tsc22d3* and by trend *Nr3c1* mRNA expression than astrocytes carrying the risk version with the A/T‐allele (orange) or wild‐type astrocytes (grey). Data points are visualized alongside with their mean ± 95% CI. Dashed lines at 0.5 and 2 delimit the area of technically irrelevant change, as determined by variability of *Sdha* expression, as this gene was used as a housekeeper to normalize for potential variation in utilized RNA concentrations

To address whether the humanization of the murine *Fkbp5* locus would interfere with the induction or suppression of other glucocorticoid response element harboring genes, *Tsc22d3*, *Nr3c1*, and *Sgk1* expression was analyzed (Figure 4). For *Tsc22d3*, the ANOVA suggested a significant interaction of strain and dexamethasone dose, *F*(8, 119) = 2.1, *p* = 0.04, for which *post hoc* was attributed to resiliency (C/G) allele carrying astrocytes after dexamethasone treatment differing from vehicle‐treated cells (1.1 ± 0.1) and resiliency (C/G) allele astrocytes exposed to 100 nM dexamethasone (25 ± 15.1) differing from wild‐type cells exposed to the same dose (10 ± 10.3). For *Sgk1* expression, only the main effect of dose was significant in the ANOVA, *F*(4, 123) = 13.5, *p* < 0.00001, which was due to astrocytes treated with 100 nM dexamethasone (2.8 ± 1.7) differing from vehicle‐treated cells (1 ± 0.3). With respect to *Nr3c1*, the ANOVA suggested a statistically significant effect of dose, *F*(4, 123) = 5.1, *p* = 0.0008, stemming from differences between vehicle‐treated (1 ± 0.2) and 100 nM dexamethasone‐treated cells (0.8 ± 0.2). Furthermore, a trend for strain, *F*(2, 123) = 2.8, *p* = 0.07, attributable to differences between wild‐type astrocytes (1 ± 0.3) and resiliency (C/G) allele carriers (0.9 ± 0.2) was found for *Nr3c1*. However, the reported changes for *Nr3c1* were within the range of technical variability.

## 4 DISCUSSION

We demonstrated that astrocytes, microglia, and neurons show a differential responsiveness to glucocorticoids based on the induction of RNA transcripts from numerous glucocorticoid‐responsive genes. In addition, we demonstrated that astrocytes, microglia, and neurons derived from mice carrying the human rs1360780 polymorphism display a differential responsiveness to glucocorticoids. Astrocytes derived from the risk (A/T) strain are more responsive than astrocytes derived from the resiliency (C/G) strain. As the SNP‐associated differences were most notable in astrocytes, our findings imply astrocytes may be a functional mediator of *FKBP5* polymorphism‐associated variations in stress responses in the CNS.

The brain is a central hub for the integration of changing internal and external conditions and, thus, plays a pivotal role in the initiation and termination of the stress response. Within this response, glucocorticoids elicit immediate transcriptional changes in their target cells. Various cells types in the brain will, dependent on their function, differently respond to glucocorticoid stimulation (Lucassen et al., 2014; Radley et al., 2015; Ressler & Smoller, 2016). Regulators of glucocorticoid signaling, such as FKBP51, will necessarily contribute to cell type‐specific responsiveness. Previous studies found a correlation of *Fkbp5* expression levels and stress responses in peripheral blood mononuclear cells and immortalized lympohblastoid cell lines (Chun et al., 2011; Yeo et al., 2017). These cell types are informative on peripheral responses of the immune system to stress. A critical missing factor in the literature was whether *Fkbp5* expression levels in the brain might affect cellular stress responsiveness. Data on human‐induced pluripotent stem cells that were differentiated into forebrain‐lineage neural cultures and exposed to a high dose of dexamethasone did not indicate a neuronal response to glucocorticoids (Liebermann et al., 2017). This is in line with our data, where neurons appeared to be protected from glucocorticoid signaling and only showed a minor induction of glucocorticoid‐responsive genes. In conformity with the common understanding of *Fkbp5* acting as potent negative regulator of glucocorticoid signaling, this low responsiveness of neurons to glucocorticoids was correlated with a high expression of *Fkbp5* at baseline in neurons. The previous remark that forebrain‐lineage neurons may not be an optimal neural cell type to examine relationships between glucocorticoid receptor activation and *Fkbp5* expression (Liebermann et al., 2017) could, thus, be extended to the hypothesis that neurons, in general, are not the primary target cells of glucocorticoid signaling. As at individual cell level the responsiveness of microglia and especially astrocytes to glucocorticoids was much stronger than in neurons, our data support the need for further study on the impact of glial FKBP51 signaling on central stress responses.

The observed prominent role of astrocytes fits with transcriptomic studies where an astrocyte‐specific gene up‐regulation after glucocorticoid exposure was reported to occur prior to adaptations in other cell types (Carter et al., 2012). This may be necessary to prevent neuronal loss by ensuring metabolic support for example via shuttling lactate (Genc et al., 2011; Ma et al., 2020) to circumvent the glucose‐restricting effects of glucocorticoids (Homer et al., 1990; Virgin et al., 1991). Our data provide insights that astrocytes could fulfill this function by promptly up‐regulating *Fkbp5* to inhibit glucocorticoid signaling and *Sgk1*, a kinase favoring cellular glucose uptake from the circulation (Boini et al., 2006). In addition, astrocytes responded to glucocorticoids by induction of *Tsc22d3*, which drives metabolism toward oxidative phosphorylation (Andre et al., 2017). This might be a means to provide astrocytes with more energy for clearance of the synaptic cleft. Notably, the combined exposure to corticosterone and the excitatory neurotransmitter glutamate was reported to deprive cells from energy in the shape of adenosine‐tri‐phosphate (Tombaugh & Sapolsky, 1992). This simultaneous presence is likely to occur during the stress response and would threat neuronal energy supply. Enhanced clearance of the synaptic cleft from glutamate by astrocytes could, therefore, be another way to safeguard neurons from shortcomings in energy.

Besides their supportive role in terms of metabolism, astrocytes were shown to influence information processing and cognition by integrating local sensory information and behavioral state (Hallmann et al., 2017; Lima et al., 2014; Oksanen et al., 2019; Peteri et al., 2019; Slezak et al., 2019). In response to glucocorticoids, resetting of the circadian clock via non‐canonical pathways (Suchmanova et al., 2019) was reported. Our data on the up‐regulation of *Per1* following stimulation with glucocorticoids suggest an astrocytic involvement in the regulation of the circadian rhythm. Furthermore, astrocytic responses to glucocorticoids were reported to directly influence (emotional) learning by structurally reorganizing neuronal networks via an increased expression of *Sgk1* (Slezak et al., 2013) or micro‐RNAs, described to influence neurogenesis (Luarte et al., 2017). Astrocytes might, hence, play an important role in shaping plasticity in response to emotional stress and set the stage for future stressful encounters (Bender et al., 2016; Tertil et al., 2018). Taken together, the observed astrocytic responses would be appropriately placed to at least partially mediate the known interaction of FKBP5 and early‐life trauma.

Very little is known about how genetic risk factors for stress vulnerability might affect astrocyte functions. By humanizing the murine *Fkbp5*‐locus with either the risk (A/T) or the resiliency (C/G) version of rs1360780, we sought to create a unique model that would allow further mechanistic studies into how these SNPs can alter the trajectory of pathology due to environmental stressors. In astrocytes, we found the risk (A/T) allele to stronger respond to glucocorticoid exposure than the resiliency (C/G) allele. The associated inherent higher reactivity of astrocytes carrying the risk rs1360780‐A/T gene variant could lead to excessive inhibition of further GC signaling, or faster inhibition via the fast intracellular feedback. These data are supported by a preliminary, albeit un‐quantified, observation that after stimulation with dexamethasone, the FKBP51 protein is also translated in the humanized mice. Taken together, this is the first demonstration that human risk and resilience *FKBP5* polymorphisms can be transferred successfully to mice.

There are technical considerations relevant to production of such humanized mouse lines. It is technically difficult to humanize a large (>16,000 bp) gene, hence, only truncated versions were inserted in place of the murine *Fkbp5*. The observed responsiveness suggests that humanized alleles can interact normally with murine gene regulator elements. Nonetheless, our results on cellular glucocorticoid responsiveness are limited to mRNA expression and quantification of cell type–specific effects on protein expression remains challenging. The SNP is only known to affect chromatin folding, not the structure or function of the protein. However, it would be important to establish normal FKBP51 interactions with the glucocorticoid receptor, such as the ligand binding affinity. Additionally, in vitro experiments can only reflect certain in vivo aspects and, hence, further studies are needed to draw conclusions as to whether the risk (A/T) allele would have functional influence on global glucocorticoid responsiveness in these mice and whether these could be used to model disorders. These mice have been made commercially available by Taconic Biosciences to aid in future studies by all researchers.

*FKBP5* SNPs have been shown to impart variability in HPA‐axis responses in the healthy population, but more importantly to impart differential risk for pathology when combined with environmental risk factors such as early life adversity. Early life adversity has been discussed to prime the stress response systems to pathological processes (Ganguly & Brenhouse, 2015; Jens & Zakreski, 2015; Kompier et al., 2019; Lesuis et al., 2018, 2019; Vaiserman & Koliada, 2017) and astrocytes were reported to be involved in the early programming of stress responsivity (Abbink et al., 2019; Gunn et al., 2013; Saavedra et al., 2017). During neuronal development, demethylation of the *FKBP5* locus as consequence of glucocorticoid exposure was reported (Provençal et al., 2019) and if the same holds true for astrocytes, allele‐specific methylation changes in *FKBP5* could exacerbate the observed *FKBP5*‐SNP‐associated differences in glucocorticoid reactivity. These data provide a first indication that differential stress sensitivity via astrocytic signaling could play a role in pathology linked to *FKBP5* SNPs.

In sum, cell type‐specific expression of *FKBP5* regulates responsiveness to glucocorticoids in the CNS with astrocytes being a promising cellular target for advancing the current knowledge on how metabolic processes and astrocytic functioning can shape the termination of the central stress response. These novel *FKBP5*‐humanized mouse lines are a unique tool to advance the current knowledge on how *FKBP5* variants interact with glucocorticoid sensitivity to influence physiology and we hope that their use in future studies will benefit patients.

## CONFLICTS OF INTEREST

Nadine Richter, Bastian Hengerer, and Kelly Allers are employees of Boehringer Ingelheim Pharma GmbH & Co KG. Verena Nold and Iris‐Tatjana Kolassa have no conflicts of interest to declare.

## AUTHOR CONTRIBUTIONS

V Nold – conception, experimental design, acquisition, analysis and interpretation of data; writing of the manuscript. N Richter – acquisition of data. B Hengerer – conception, revision of the manuscript. IT Kolassa – data interpretation, revision of the manuscript. KA Allers – conception, data interpretation, revision of manuscript.

### PEER REVIEW

The peer review history for this article is available at https://publons.com/publon/10.1111/ejn.14999.

## Supporting information

Supplementary Material

Supplementary Material

Supplementary Material

Supplementary Material

## Data Availability

All raw data files, summary data frames, and the R code written to analyze and visualize the herein contained data will be made available by the corresponding author upon request.

## References

[ejn14999-bib-0001] Abbink, M. R. , van Deijk, A.‐L.‐ F. , Heine, V. M. , Verheijen, M. H. , & Korosi, A. (2019). The involvement of astrocytes in early‐life adversity induced programming of the brain. Glia, 67(9), 1637–1653. 10.1002/glia.23625 31038797 PMC6767561

[ejn14999-bib-0002] Andre, F. , Trinh, A. , Balayssac, S. , Maboudou, P. , Dekiouk, S. , Malet‐Martino, M. , Quesnel, B. , Idziorek, T. , Kluza, J. , & Marchetti, P. (2017). Metabolic rewiring in cancer cells overexpressing the glucocorticoid‐induced leucine zipper protein (GILZ): Activation of mitochondrial oxidative phosphorylation and sensitization to oxidative cell death induced by mitochondrial targeted drugs. The International Journal of Biochemistry & Cell Biology, 85, 166–174. 10.1016/j.biocel.2017.02.011 28259749

[ejn14999-bib-0003] Appel, K. , Schwahn, C. , Mahler, J. , Schulz, A. , Spitzer, C. , Fenske, K. , Stender, J. , Barnow, S. , John, U. , Teumer, A. , Biffar, R. , Nauck, M. , Völzke, H. , Freyberger, H. J. , & Grabe, H. J. (2011). Moderation of adult depression by a polymorphism in the FKBP5 gene and childhood physical abuse in the general population. Neuropsychopharmacology, 36(10), 1982–1991. 10.1038/npp.2011.81 21654733 PMC3158316

[ejn14999-bib-0004] Bender, C. L. , Calfa, G. D. , & Molina, V. A. (2016). Astrocyte plasticity induced by emotional stress: A new partner in psychiatric physiopathology? Progress in Neuro‐Psychopharmacology & Biological Psychiatry, 65, 68–77. 10.1016/j.pnpbp.2015.08.005 26320029

[ejn14999-bib-0005] Binder, E. B. (2009). The role of FKBP5, a co‐chaperone of the glucocorticoid receptor in the pathogenesis and therapy of affective and anxiety disorders. Psychoneuroendocrinology, 34(Suppl 1), S186–S195. 10.1016/j.psyneuen.2009.05.021 19560279

[ejn14999-bib-0006] Binder, E. B. , Bradley, R. G. , Liu, W. , Epstein, M. P. , Deveau, T. C. , Mercer, K. B. , Tang, Y. , Gillespie, C. F. , Heim, C. M. , Nemeroff, C. B. , Schwartz, A. C. , Cubells, J. F. , & Ressler, K. J. (2008). Association of FKBP5 polymorphisms and childhood abuse with risk of posttraumatic stress disorder symptoms in adults. JAMA, 299(11), 1291–1305. 18349090 10.1001/jama.299.11.1291 PMC2441757

[ejn14999-bib-0007] Boini, K. M. , Hennige, A. M. , Huang, D. Y. , Friedrich, B. , Palmada, M. , Boehmer, C. , Grahammer, F. , Artunc, F. , Ullrich, S. , Avram, D. , Osswald, H. , Wulff, P. , Kuhl, D. , Vallon, V. , Haring, H.‐U. , & Lang, F. (2006). Serum‐ and glucocorticoid‐inducible kinase 1 mediates salt sensitivity of glucose tolerance. Diabetes, 55(7), 2059–2066. 10.2337/db05-1038 16804076

[ejn14999-bib-0009] Carter, B. S. , Meng, F. , & Thompson, R. C. (2012). Glucocorticoid treatment of astrocytes results in temporally dynamic transcriptome regulation and astrocyte‐enriched mRNA changes in vitro. Physiological Genomics, 44(24), 1188–1200. 10.1152/physiolgenomics.00097.2012 23110767 PMC3544487

[ejn14999-bib-0010] Chandola, T. , Brunner, E. , & Marmot, M. (2006). Chronic stress at work and the metabolic syndrome: Prospective study. BMJ, 332(7540), 521–525. 10.1136/bmj.38693.435301.80 16428252 PMC1388129

[ejn14999-bib-0011] Chun, E. , Lee, H. S. , Bang, B. R. , Kim, T. W. , Lee, S. H. , Kim, J. H. , Cho, S.‐H. , Min, K.‐U. , Kim, Y.‐Y. , & Park, H.‐W. (2011). Dexamethasone‐induced FKBP51 expression in peripheral blood mononuclear cells could play a role in predicting the response of asthmatics to treatment with corticosteroids. Journal of Clinical Immunology, 31(1), 122–127. 20853021 10.1007/s10875-010-9463-9

[ejn14999-bib-0013] Cohen, S. , Janicki‐Deverts, D. , Doyle, W. J. , Miller, G. E. , Frank, E. , Rabin, B. S. , & Turner, R. B. (2012). Chronic stress, glucocorticoid receptor resistance, inflammation, and disease risk. Proceedings of the National Academy of Sciences of the United States of America, 109(16), 5995–5999. 10.1073/pnas.1118355109 22474371 PMC3341031

[ejn14999-bib-0014] Criado‐Marrero, M. , Gebru, N. T. , Gould, L. A. , Smith, T. M. , Kim, S. , Blackburn, R. J. , Dickey, C. A. , & Blair, L. J. (2019). Early life stress and high FKBP5 interact to increase anxiety‐like symptoms through altered AKT signaling in the dorsal hippocampus. International Journal of Molecular Sciences, 20(11), 2738 10.3390/ijms20112738 PMC6600369 31167373

[ejn14999-bib-0015] Fries, G. R. , Gassen, N. C. , & Rein, T. (2017). The FKBP51 glucocorticoid receptor co‐chaperone: Regulation, function, and implications in health and disease. International Journal of Molecular Sciences, 18(12), 2614. 10.3390/ijms18122614 PMC5751217 29206196

[ejn14999-bib-0016] Ganguly, P. , & Brenhouse, H. C. (2015). Broken or maladaptive? Altered trajectories in neuroinflammation and behavior after early life adversity. Developmental Cognitive Neuroscience, 11, 18–30. 10.1016/j.dcn.2014.07.001 25081071 PMC4476268

[ejn14999-bib-0018] Genc, S. , Kurnaz, I. A. , & Ozilgen, M. (2011). Astrocyte‐neuron lactate shuttle may boost more ATP supply to the neuron under hypoxic conditions–in silico study supported by in vitro expression data. BMC Systems Biology, 5, 162 10.1186/1752-0509-5-162 21995951 PMC3202240

[ejn14999-bib-0019] Gunn, B. G. , Cunningham, L. , Cooper, M. A. , Corteen, N. L. , Seifi, M. , Swinny, J. D. , Lambert, J. J. , & Belelli, D. (2013). Dysfunctional astrocytic and synaptic regulation of hypothalamic glutamatergic transmission in a mouse model of early‐life adversity: Relevance to neurosteroids and programming of the stress response. The Journal of Neuroscience, 33(50), 19534–19554. 10.1523/JNEUROSCI.1337-13.2013 24336719 PMC3858624

[ejn14999-bib-0020] Hallmann, A.‐L. , Araúzo‐Bravo, M. J. , Mavrommatis, L. , Ehrlich, M. , Röpke, A. , Brockhaus, J. , Missler, M. , Sterneckert, J. , Schöler, H. R. , Kuhlmann, T. , Zaehres, H. , & Hargus, G. (2017). Astrocyte pathology in a human neural stem cell model of frontotemporal dementia caused by mutant TAU protein. Scientific Reports, 7(1), 42991 10.1038/srep42991 28256506 PMC5335603

[ejn14999-bib-0021] Hohne, N. , Poidinger, M. , Merz, F. , Pfister, H. , Bruckl, T. , Zimmermann, P. , Uhr, M. , Holsboer, F. , & Ising, M. (2014). FKBP5 genotype‐dependent DNA methylation and mRNA regulation after psychosocial stress in remitted depression and healthy controls. The International Journal of Neuropsychopharmacology, 18(4), pyu087 10.1093/ijnp/pyu087 25522420 PMC4360217

[ejn14999-bib-0022] Homer, H. C. , Packan, D. R. , & Sapolsky, R. M. (1990). Glucocorticoids inhibit glucose transport in cultured hippocampal neurons and glia. Neuroendocrinology, 52(1), 57–64. 10.1159/000125539 2118608

[ejn14999-bib-0023] Ising, M. , Depping, A. M. , Siebertz, A. , Lucae, S. , Unschuld, P. G. , Kloiber, S. , Horstmann, S. , Uhr, M. , Mller‐Myhsok, B. , & Holsboer, F. (2008). Polymorphisms in the FKBP5 gene region modulate recovery from psychosocial stress in healthy controls. The European Journal of Neuroscience, 28(2), 389–398. 10.1111/j.1460-9568.2008.06332.x 18702710

[ejn14999-bib-0024] Jaaskelainen, T. , Makkonen, H. , & Palvimo, J. J. (2011). Steroid up‐regulation of FKBP51 and its role in hormone signaling. Current Opinion in Pharmacology, 11(4), 326–331. 21531172 10.1016/j.coph.2011.04.006

[ejn14999-bib-0025] Jens, P. , & Zakreski, E. (2015). The programming of the stress response network by early‐life adversity. Psychoneuroendocrinology, 61, 11.

[ejn14999-bib-0026] Juster, R. P. , McEwen, B. S. , & Lupien, S. J. (2010). Allostatic load biomarkers of chronic stress and impact on health and cognition. Neuroscience and Biobehavioral Reviews., 35(1), 2–16. 10.1016/j.neubiorev.2009.10.002 19822172

[ejn14999-bib-0028] Klengel, T. , Mehta, D. , Anacker, C. , Rex‐Haffner, M. , Pruessner, J. C. , Pariante, C. M. , Pace, T. W. W. , Mercer, K. B. , Mayberg, H. S. , Bradley, B. , Nemeroff, C. B. , Holsboer, F. , Heim, C. M. , Ressler, K. J. , Rein, T. , & Binder, E. B. (2013). Allele‐specific FKBP5 DNA demethylation mediates gene‐childhood trauma interactions. Nature Neuroscience, 16(1), 33–41. 10.1038/nn.3275 23201972 PMC4136922

[ejn14999-bib-0029] Kompier, N. F. , Keysers, C. , Gazzola, V. , Lucassen, P. J. , & Krugers, H. J. (2019). Early life adversity and adult social behavior: Focus on arginine vasopressin and oxytocin as potential mediators. Frontiers in Behavioral Neuroscience, 13, 143 10.3389/fnbeh.2019.00143 31404254 PMC6676334

[ejn14999-bib-0030] Lagraauw, H. M. , Kuiper, J. , & Bot, I. (2015). Acute and chronic psychological stress as risk factors for cardiovascular disease: Insights gained from epidemiological, clinical and experimental studies. Brain, Behavior, and Immunity, 50, 18–30. 10.1016/j.bbi.2015.08.007 26256574

[ejn14999-bib-0031] Lesuis, S. L. , Hoeijmakers, L. , Korosi, A. , de Rooij, S. R. , Swaab, D. F. , Kessels, H. W. , Lucassen, P. J. , & Krugers, H. J. (2018). Vulnerability and resilience to Alzheimer's disease: Early life conditions modulate neuropathology and determine cognitive reserve. Alzheimer's Research & Therapy, 10(1), 95 10.1186/s13195-018-0422-7 PMC6145191 30227888

[ejn14999-bib-0032] Lesuis, S. L. , Lucassen, P. J. , & Krugers, H. J. (2019). Early life stress amplifies fear responses and hippocampal synaptic potentiation in the APPswe/PS1dE9 Alzheimer mouse model. Neuroscience. 10.1016/j.neuroscience.2019.07.012 31302265

[ejn14999-bib-0033] Liebermann, R. , Kranzler, H. R. , Levine, E. S. , & Covault, J. (2017). Examining FKBP5 mRNA expression in human iPSC‐derived neural cells. Psychiatry Research, 247, 172–181. 10.1016/j.psychres.2016.11.027 27915167 PMC5191911

[ejn14999-bib-0034] Lima, A. , Sardinha, V. M. , Oliveira, A. F. , Reis, M. , Mota, C. , Silva, M. A. , Marques, F. , Cerqueira, J. J. , Pinto, L. , Sousa, N. , & Oliveira, J. F. (2014). Astrocyte pathology in the prefrontal cortex impairs the cognitive function of rats. Molecular Psychiatry, 19(7), 834–841. 10.1038/mp.2013.182 24419043

[ejn14999-bib-0035] Lorenz, O. R. , Freiburger, L. , Rutz, D. A. , Krause, M. , Zierer, B. K. , Alvira, S. , Cuéllar, J. , Valpuesta, J. M. , Madl, T. , Sattler, M. , & Buchner, J. (2014). Modulation of the Hsp90 chaperone cycle by a stringent client protein. Molecular Cell, 53(6), 941–953. 24613341 10.1016/j.molcel.2014.02.003

[ejn14999-bib-0036] Luarte, A. , Cisternas, P. , Caviedes, A. , Batiz, L. F. , Lafourcade, C. , Wyneken, U. , & Henzi, R. (2017). Astrocytes at the hub of the stress response: Potential modulation of neurogenesis by miRNAs in astrocyte‐derived exosomes. Stem Cells International, 2017, 1–13. 10.1155/2017/1719050 PMC5610870 29081809

[ejn14999-bib-0037] Lucassen, P. J. , Pruessner, J. , Sousa, N. , Almeida, O. F. , Van Dam, A. M. , Rajkowska, G. , Swaab, D. F. , & Czéh, B. (2014). Neuropathology of stress. Acta Neuropathologica, 127(1), 109–135. 24318124 10.1007/s00401-013-1223-5 PMC3889685

[ejn14999-bib-0038] Ma, R. D. , Zhou, G. J. , Qu, M. , Yi, J. H. , Tang, Y. L. , Yang, X. Y. , Nie, Y.‐X. , & Gu, H.‐F. (2020). Corticosterone induces neurotoxicity in PC12 cells via disrupting autophagy flux mediated by AMPK/mTOR signaling. CNS Neuroscience & Therapeutics, 26(2), 167–176. 10.1111/cns.13212 31423743 PMC6978254

[ejn14999-bib-0039] Machado, A. , Herrera, A. J. , de Pablos, R. M. , Espinosa‐Oliva, A. M. , Sarmiento, M. , Ayala, A. , Venero, J. L. , Santiago, M. , Villarán, R. F. , Delgado‐Cortés, M. J. , Argüelles, S. , & Cano, J. (2014). Chronic stress as a risk factor for Alzheimer's disease. Reviews in the Neurosciences, 25(6), 785–804. 10.1515/revneuro-2014-0035 25178904

[ejn14999-bib-0040] McEwen, B. S. (2004). Protection and damage from acute and chronic stress: Allostasis and allostatic overload and relevance to the pathophysiology of psychiatric disorders. Annals of the New York Academy of Sciences., 1032, 1–7. 10.1196/annals.1314.001 15677391

[ejn14999-bib-0043] Nold, V. , Sweatman, C. , Karabatsiakis, A. , Boeck, C. , Bretschneider, T. , Lawless, N. , Fundel‐Clemens, K. , Kolassa, I.‐T. , & Allers, K. (2019). Activation of the kynurenine pathway and mitochondrial respiration to face allostatic load in a double‐hit model of stress. Psychoneuroendocrinology., 107, 148–159. 10.1016/j.psyneuen.2019.04.006 31129488

[ejn14999-bib-0044] Oksanen, M. , Lehtonen, S. , Jaronen, M. , Goldsteins, G. , Hämäläinen, R. H. , & Koistinaho, J. (2019). Astrocyte alterations in neurodegenerative pathologies and their modeling in human induced pluripotent stem cell platforms. Cellular and Molecular Life Sciences, 76(14), 2739–2760. 10.1007/s00018-019-03111-7 31016348 PMC6588647

[ejn14999-bib-0046] Pariante, C. M. (2009). Risk factors for development of depression and psychosis. Glucocorticoid receptors and pituitary implications for treatment with antidepressant and glucocorticoids. Annals of the New York Academy of Sciences, 1179, 144–152. 10.1111/j.1749-6632.2009.04978.x 19906237 PMC2982725

[ejn14999-bib-0047] Perrin, A. J. , Horowitz, M. A. , Roelofs, J. , Zunszain, P. A. , & Pariante, C. M. (2019). Glucocorticoid resistance: Is it a requisite for increased cytokine production in depression? A systematic review and meta‐analysis. Frontiers in Psychiatry, 10, 423 10.3389/fpsyt.2019.00423 31316402 PMC6609575

[ejn14999-bib-0048] Peteri, U.‐K. , Niukkanen, M. , & Castrén, M. L. (2019). Astrocytes in neuropathologies affecting the frontal cortex. Frontiers in Cellular Neuroscience, 13, 44 10.3389/fncel.2019.00044 30809131 PMC6379461

[ejn14999-bib-0049] Pirkl, F. , & Buchner, J. (2001). Functional analysis of the Hsp90‐associated human peptidyl prolyl cis/trans isomerases FKBP51, FKBP52 and Cyp40. Journal of Molecular Biology, 308(4), 795–806. 11350175 10.1006/jmbi.2001.4595

[ejn14999-bib-0050] Provençal, N. , Arloth, J. , Cattaneo, A. , Anacker, C. , Cattane, N. , Wiechmann, T. , Röh, S. , Ködel, M. , Klengel, T. , Czamara, D. , Müller, N. S. , Lahti, J. , PREDO team , Räikkönen, K. , Pariante, C. M. , & Binder, E. B. (2019). Glucocorticoid exposure during hippocampal neurogenesis primes future stress response by inducing changes in DNA methylation. Proceedings of the National Academy of Sciences of the United States of America, 117, 23280–23285. 10.1016/j.psyneuen.2019.07.212 31399550 PMC7519233

[ejn14999-bib-0051] Radley, J. , Morilak, D. , Viau, V. , & Campeau, S. (2015). Chronic stress and brain plasticity: Mechanisms underlying adaptive and maladaptive changes and implications for stress‐related CNS disorders. Neuroscience and Biobehavioral Reviews, 58, 79–91. 26116544 10.1016/j.neubiorev.2015.06.018 PMC4684432

[ejn14999-bib-0053] Rauch, S. L. , Shin, L. M. , & Phelps, E. A. (2006). Neurocircuitry models of posttraumatic stress disorder and extinction: Human neuroimaging research—Past, present, and future. Biological Psychiatry, 60(4), 376–382. 10.1016/j.biopsych.2006.06.004 16919525

[ejn14999-bib-0054] Ressler, K. J. , & Smoller, J. W. (2016). Impact of stress on the brain: Pathology, treatment and prevention. Neuropsychopharmacology, 41(1), 1–2. 26657948 10.1038/npp.2015.306 PMC4677154

[ejn14999-bib-0055] Saavedra, L. M. , Fenton Navarro, B. , & Torner, L. (2017). Early life stress activates glial cells in the hippocampus but attenuates cytokine secretion in response to an immune challenge in rat pups. NeuroImmunoModulation, 24(4–5), 242–255. 10.1159/000485383 29332092

[ejn14999-bib-0056] Scammell, J. G. , Hubler, T. R. , Denny, W. B. , & Valentine, D. L. (2003). Organization of the human FK506‐binding immunophilin FKBP52 protein gene (FKBP4). Genomics, 81(6), 640–643. 12782134 10.1016/s0888-7543(03)00090-9

[ejn14999-bib-0058] Slezak, M. , Kandler, S. , Van Veldhoven, P. P. , Van den Haute, C. , Bonin, V. , & Holt, M. G. (2019). Distinct mechanisms for visual and motor‐related astrocyte responses in mouse visual cortex. Current Biology, 29(18), 3120–3127.e5. 10.1016/j.cub.2019.07.078 31495587 PMC6859477

[ejn14999-bib-0059] Slezak, M. , Korostynski, M. , Gieryk, A. , Golda, S. , Dzbek, J. , Piechota, M. , Wlazlo, E. , Bilecki, W. , & Przewlocki, R. (2013). Astrocytes are a neural target of morphine action via glucocorticoid receptor‐dependent signaling. Glia, 61(4), 623–635. 10.1002/glia.22460 23339081

[ejn14999-bib-0060] Suchmanova, K. , Sladek, M. , Cecmanova, V. , Shrestha, T. C. , Ralph, M. R. , & Sumova, A. (2019). Dexamethasone resets the circadian clock in hippocampus via multiple mechanisms involving lithium‐independent GSK3beta signalling. British Journal of Pharmacology, 177, 4074. 10.1111/bph.14834 PMC7429476 31423567

[ejn14999-bib-0062] Tertil, M. , Skupio, U. , Barut, J. , Dubovyk, V. , Wawrzczak‐Bargiela, A. , Soltys, Z. , Golda, S. , Kudla, L. , Wiktorowska, L. , Szklarczyk, K. , Korostynski, M. , Przewlocki, R. , & Slezak, M. (2018). Glucocorticoid receptor signaling in astrocytes is required for aversive memory formation. Translational Psychiatry, 8(1), 255 10.1038/s41398-018-0300-x 30487639 PMC6261947

[ejn14999-bib-0063] Tombaugh, G. C. , & Sapolsky, R. M. (1992). Corticosterone accelerates hypoxia‐ and cyanide‐induced ATP loss in cultured hippocampal astrocytes. Brain Research, 588(1), 154–158. 10.1016/0006-8993(92)91356-J 1356586

[ejn14999-bib-0066] Touma, C. , Gassen, N. C. , Herrmann, L. , Cheung‐Flynn, J. , Bull, D. R. , Ionescu, I. A. , Heinzmann, J.‐M. , Knapman, A. , Siebertz, A. , Depping, A.‐M. , Hartmann, J. , Hausch, F. , Schmidt, M. V. , Holsboer, F. , Ising, M. , Cox, M. B. , Schmidt, U. , & Rein, T. (2011). FK506 binding protein 5 shapes stress responsiveness: Modulation of neuroendocrine reactivity and coping behavior. Biological Psychiatry, 70(10), 928–936. 10.1016/j.biopsych.2011.07.023 21907973

[ejn14999-bib-0068] Vaiserman, A. M. , & Koliada, A. K. (2017). Early‐life adversity and long‐term neurobehavioral outcomes: Epigenome as a bridge? Human Genomics, 11(1), 34 10.1186/s40246-017-0129-z 29246185 PMC5732459

[ejn14999-bib-0069] Virgin, C. E. Jr , Ha, T. P. , Packan, D. R. , Tombaugh, G. C. , Yang, S. H. , Horner, H. C. , & Sapolsky, R. M. (1991). Glucocorticoids inhibit glucose transport and glutamate uptake in hippocampal astrocytes: Implications for glucocorticoid neurotoxicity. Journal of Neurochemistry, 57(4), 1422–1428. 10.1111/j.1471-4159.1991.tb08309.x 1680166

[ejn14999-bib-0070] Wilker, S. , Pfeiffer, A. , Kolassa, S. , Elbert, T. , Lingenfelder, B. , Ovuga, E. , Papassotiropoulos, A. , de Quervain, D. , & Kolassa, I.‐T. (2014). The role of FKBP5 genotype in moderating long‐term effectiveness of exposure‐based psychotherapy for posttraumatic stress disorder. Translational Psychiatry, 4, e403 10.1038/tp.2014.49 24959896 PMC4080328

[ejn14999-bib-0071] Wochnik, G. M. , Ruegg, J. , Abel, G. A. , Schmidt, U. , Holsboer, F. , & Rein, T. (2005). FK506‐binding proteins 51 and 52 differentially regulate dynein interaction and nuclear translocation of the glucocorticoid receptor in mammalian cells. The Journal of Biological Chemistry, 280(6), 4609–4616. 15591061 10.1074/jbc.M407498200

[ejn14999-bib-0072] Yeo, S. , Enoch, M. A. , Gorodetsky, E. , Akhtar, L. , Schuebel, K. , Roy, A. , & Goldman, D. (2017). The influence of FKBP5 genotype on expression of FKBP5 and other glucocorticoid‐regulated genes, dependent on trauma exposure. Genes, Brain and Behaviour, 16(2), 223–232. 10.1111/gbb.12342 PMC6233292 27648526

[ejn14999-bib-0074] Zimmermann, P. , Bruckl, T. , Nocon, A. , Pfister, H. , Binder, E. B. , Uhr, M. , Lieb, R. , Moffitt, T. E. , Caspi, A. , Holsboer, F. , Ising, M. (2011). Interaction of FKBP5 gene variants and adverse life events in predicting depression onset: Results from a 10‐year prospective community study. The American Journal of Psychiatry, 168(10), 1107–1116. 21865530 10.1176/appi.ajp.2011.10111577 PMC3856576

