# Supplementary material for: FKBP5 polymorphisms induce differential glucocorticoid responsiveness in primary CNS cells – First insights from novel humanized mice
Source: Eur J Neurosci. 2020 Oct 27;53(2):402–15. doi: 10.1111/ejn.14999 (PMC7894319; doi:10.1111/ejn.14999)
Supplement: Supplementary file 1 — Supplementary Material [file EJN-53-402-s001.docx]

**Supplements**


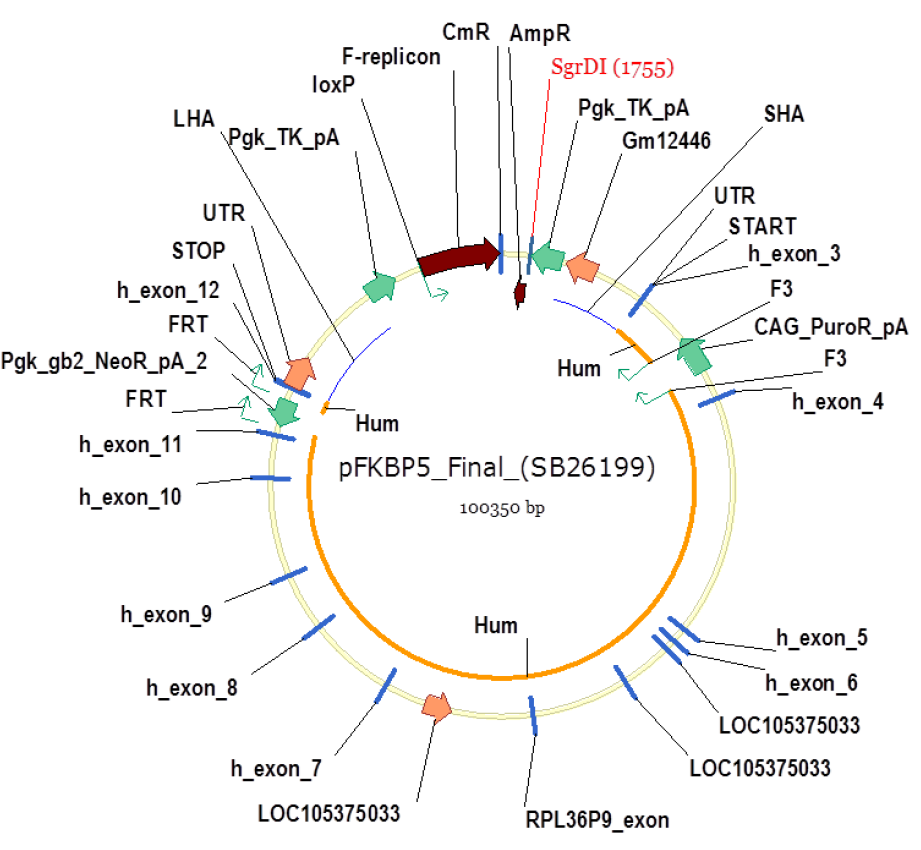


**Fig. A1.** Schematic overview of the targeting vector used for the generation of the *Fkbp5*-humanized mouse strains at Taconic Biosciences. SgrDI was used for linearization.

**Text A1.** Sequence of the *Fkbp5* locus in mouse strain C57BL/6NTac-Fkbp5tm4570 (FKBP5) Tac, carrying the risk-associated rs1360780-A/T and the mouse line C57BL/6NTac-Fkbp5tm4571 (FKBP5) Tac carrying the resiliency (C/G) version. The point mutation at position 3622 is marked in red and bigger font. No other differences within the locus were detected by sequencing.

Acacatcccgtgttctacaagaccctttatctaccaggagtgcttcataaatatccacatccagctgagaagagctaaggaagggtaaaatggcaaaacgacttgcatgggcattgtggatctggctcatgagagaaagaagagagagtaatcaaatgggtgtggctggttatggaaagggagctggttggtttgaccagtgtgtgtgaaggagagtggcagaacaccagaggcctctgttctctctcaacaatatggctgtaggagaagacttgttttatggttctgagagggcagcttctccctgtgttgtcctgttaaggaatataattaccggcaggcctgacttatactgctctcatctaggaccgtggctagacgggaaattgaagaagtgggtgatgttttatttccttgtctgatggcatcaatgcatgcccacagcaaatgcttttgttattgtttctgtgttttaactctattagtcttatgtaagcttagttctcagggacttttcaggtgtgttaagaagtgatactttgattgaaggaaatgcatttctcttggcaggtcttctacttacaaaggacaatgactactgatgaaggtgccaagaacaatgaagaaagccccacagccactgttgctgagcagggagaggatattacctccaaaaaagacaggggagtattaaaggtgaggccacaaagctgagtgacatacaaggaaacctgctgtctggaaagattatagcatctctttctgaggggggctggggtggataatgtttattgatggttactaagtgccaaacactagagctagattctcattttgtcttcataacttcaaggttttttttaagagttgagtctcgttatgttgcccaggctggtcttgagctcctgggttcaagtgatcttcccatctcagcttcctgagtagctgggattttctttttaaattaattaattaaatttttgagagagggcatctcattatgttgctcaggcaagtcttgaaatcctggcctcaagcaatcctcccacctcagccttctgagtagctgggattataggcacgagccactgtgcccagctagagattttaatttttaatcccagattatagatgttgaaattgagactgggctaggtaaattaacttgcctaatatcatatatcttagtaatgcagatttacctggggttcaggtccaagttatatctcactctaaagggctatatttccctattctcagagtatacaaataggttactattttaatgtatccttttagtacactaaagctagctaatgacaattttgaagactttttaaggaaagaaaagctaacttgaaaaattcaaaggggttttattttattttattttattttattttattttattttattttattttgagacagagtctccctctgttgcctgggctagagtgcagtggtatgatctcagctcactgcaacctccgcctcctggattcaagcaattctgcctcagcctcccgagtagctgggattacaggcgcctgctaccatgcctcgctaatttttgtagagacagggtttcaccatgttggccaggctggtcttgaactcctgacctcaggtgatccgcctgccttggcctcgcaaagtgttgggattacaggcatgagccaccgtgcccggcccaaaggggtttatttttatatataaatattgttcctgtgtacagtgaagatatcttttgcgatttcctcttagaatatatcttacttttgaattagctttcctacttattaaagcactatacagtcattcaccatataatgacatttcagtcagtgacagactgcaccttcagcagtggtcccataagattataatggggccgaaaagttcctatggcctagtggtgccatagctgttgtagcatgggtagtacagtgtattatgtgtttgtggtgatgtgggtgtaaacaaacctactgcactgcagtcacagttatgtatagtacataatacttgataataaatgtttatattactggtttatgtgtttactatactttctatcattattttagagtgtgcttcttattttttaaaaagttaactgtaaaacacccttggctgagtgcagtggctcatgcctgtaatcccagcaccctgggaggccaaagcaagaggatcacttgaggccaggagttcaagaccagcctgggcaacatagtaagacccctatctgtagaaaaaaaattaaaaaaattagccaggcatagtggcatgtacctgtagtttctactactcaggagactgaggcaggaggatcatgtaagcctgggagttccaagctgcagtgaactatgatcataccactgcattctagcctgggtgagagtgagatcctgtctcaaaaacaaatgaacaaacaaaaaacagccttgggcaggtccctcaggaggtattcaagaagaaggcattgttattataggagacagcagctccatgcatgttactgtcctgaagaccttccagtgggacaagatgtgttggtggaagacagtgatactgatgatcctgaccctctgtaggcctaggtttatgtatgtgtttgtgtcttagttttaaacaaaaaaaagtttaaaaagtaaaagaataaaaaatttaaaaaacagaaaaatgcttatagaataaggatataaagaaagaaaatatttttgtacagctgtacagtgtttgtgttctaagtgtcattacaagagtcaaagctaaaaaaaaagtttacacagtaaaaatgttatagtaagctaagattgatttattattaaagaaagaagaaaaattttaaaataaatgtagtctaggtgtccagtgtttataaagtctacggtagtgtatggtaatatcctaggccttcatgttcactcactgctcactcgctgactcatccagaacaacttccagtcctgcaagttccattcatggtaagtgttctgtataggtgtaccattttttttcttgtataccttttattactgtacctttttctatgttgagatatgcttagatacacaaataccactgttttacaattgcctgcagtattcagtgcagtaacacaatgtacaggtttgtagccttggagcagtaggccaaaccacctaacgtgggtgtgtacaagtaggccaggccatctaggtttgtgtaagtacactctgcgatgttcacataaggatgaaatcatctaagaacacatttctcagaacatatccctgtccttaagcgatgcatgactgtgtgtgtttcttcactctatagttaatcattttcacgtgcatttcttttgttttgtttcttacaacaatgtggtgagacaatcagagcatctcttgattcttccatcttataagtgaataaactgaatctgagaaaggttaagtggctttagtcacattaaatagttatttctcctacaccattgttaacacatgtaagttttgtatagttaagttttgtttttcctgaaaagattatctgatgcattctgaatattaccaggatgctgagttttaatatctcttgtgccagcagtagcaagtaagaatttttgttttgt**a/c**taactttgcttatgtgaaagccttctgtgcctggatctcttctgaatatatgtgaattgctattatgtgctaagtcaaattttggggacttgcagctatagaataagggctttatgctatagatttacatcctattgtctttttttagaaggcttctcaaccttgtagctatagttactcatcctaaattccaggaagcttctagaaacagtggagactttgcaggctgtcctcagcatctccctgcctctgtttacgacttaccctttcatcttttcagagtgcccatgcccagagaacatgatccccaacttgcctcagaccctgttctgttctagggagtaaatctaattgcaagatttttaggcttattaagaaacatttgagatttgaagccctttaaattatatcatttatcattgtctagagcctcggatttcaccatgaggcttttggttaagatttaaataggtctagtaactgtgcaagctagggcaatttagttaacctctctgtgattaagttcctgtttgtaaaattagaataataatgccatcaacctcatagagttattaggattaggtgaagcacttagcatagtgcctgtcctatagtaatctatcttattaactattacgtatttttggcgcgccatttaaatgaagttcctatactatttgaagaataggaacttcggaataggaacttcatttaaatggttacccctaggctcgagcctaggaaaaaaagaaccagtgctggtttggaaattcagtaactatctgaggaagataaaagatggatttatattttaaaaatgaatagaacctagattgtgttcttaaggcaactctagatttaggtacaaattcatcttgctagggaagcctgaagtgataccatagtaccacttgctggtgtggatggctctctgactctctggtagccattcatcccacatgcccagttactgtttatagtcctagaaatgcagattccttccacgtgaggcttacagttgagcatagcctcccaaataccacatgcagttcgtgaacatgagcacttgtagaatattgtgtaatatcttttagattaatgttttgagactttttttttcttttgaagtcctagaagggcagtagtgtttctttgcctttaaagaatatttggggctgggtgcgatagcttataccagtaatctcagcactttaggaggctgaggcgggagaatcattggaactcaggagttccaaaccagcctgggcaatgtagcaagatctcatctctactaaaaataaaaaaatttgccaggcgtggtggcatgtgcctgtagtctctgctactcgggaggctgaggtgggatgatcacttgagctgagtgatagaggctacagtgagctatgattgtaccaccgcatgcactctagcctgggtaaaagagcaagaccctgtctcaaaaaataaaataaagatgtttgggcttttgagttaagaaagctgtggaatttaagttcacagtgtttatgtgatatttatgttaggtggacggtatacttgtaaaaaaagaatttaggagcaaataatcgaattatttaaataaaactcaaagcaaactccttccccaaaagaaatgtgtttcatggacccttttatgcaatttatcctgttctggttacttcttgttttgttgttttttttgtctacccaactaaattataagcttccttaggacacactctatttttcaatactatttttgttctatctctgcctagaaaatttgcgcttattatagttgaaatgataaattaggcaacaattgcaacatagccatatttcatggataatgttaccaccagttttcaaagacttaatttttgagtagtttaggaatttatattactttcatccctttcagcagggaatcctttttgagacagggtctcactctgtcacccaggctagagtgcagtggcataatcatgactcactgaagcctcaatctcctgggctcccaggcttaagctgtcctcccaccacagcctccggagtagctgggactacaggtgtgcaccatacccagctgtgttctttggttctttggggtgttttttttttttttttagagatgaggtcttgctgtgttgcccaggctggtgtcaaattcctggactcaagcagtcctcccacctcagcctcccaaagtgctgggccgatcaacagggaatcttttgtgatagccttcaaaaaaaataatttatttatttgtatgtagcactctgtcatcttgtagcccatactttctagctgagtatgtagtatgaaaaactggaaattagaactttggttttatgaaagttaaatctttttaaatagggcttatagctttcaactaccttttcctttcccttaggccaagtcatcaaggcatgggacattggggtggctaccatgaagaaaggagagatatgccatttactgtgcaaaccagaatatgcatatggctcggctggcagtctccctaaaattccctcgaatgcaactctcttttttgaggcaagtatgtgtgtgatgttgccttgtcagcatctgctctacaataggaatggctttgaagcaacaggcagtgggaaaaggagctaagcttgctatagagaatcagatccaacttacttattttggagcatggcttcttgaattgtttcccacaaacccaagggtgcgcgtgccagtgctttcttagaaacgtgtttctagcacaaaaaggtcatatccaaataaacaaaagacgctacacttagaaataaagattctggtccatggtgtgagtgtgacatactcaaaattgaccagagtacaggatggaaatgttggcgataaatgttgaaatatgcctatgtcacgttgcggggtatttacatattataaaaatttatgtgtcacagatctcacttctctagaagccgttggtggtgataaaacattattcatcctatgttgaattacatatatttggcaaaacttgatttttaagaaccaccccagctaggtaaaatacacctttaatcagtcgtaggtcacatccatgagatttattcttacattttgagaagggaactccataaacccaggatgggaaagttgtggcctcaaacaaaatagttctacaccagcaccttgtagctctggctttttgcccacagggtagttttcctagtatttcctatctttgcactgcagtcccttaggatatagttctagacattagcattacatgaaattctttccaagtatggaaaagtatagtgggtttttttttttttgctacttttccaataattaagatgcataaatgaatgtatatattttaaaacagctttattttgtaaaatgaaataattggttgcccttgccttcacatatgttcagttggctttatttggtcttttctcagctgaattccttttttctttctagattgagctccttgatttcaaaggagaggatttatttgaagatggaggcattatccggagaaccaaacggaaaggagagggatattcaaatccaaacgaaggagcaacagtagaaagtaagtattaaagtaagagggaactagtccttaaaagttgggccatttatgtggacgaaatggagacttaaaaaaaaatgtttgtagaaatgaatatatcggctgtgcgcagtgactcacgcctgtaatcccagcactttgggaggtcgaggcgggtggattgcctgagctcaggagtttgagaccagcctggccagcatggcgaaaccccatctctactaaaaatacaaaaaactagccaggtgtggtggtgcacgcctgtattcccagctactcgggaggctgaggcatgagaatcacttgaacccgggaggcaggggttgcagtgagtcgagatggtgctactgcactccagcctggatgacaaagcaagactctgtctcaaaaaaaaagtattgaatatatctcaaagatagtcttagctgggcacagtggctcatgcctgtaattccagcactttgggaggccgaggcaggaggatctcttgagcttaggaattcgagaccagccaggacagcatagtgaggccccatctctactaaaaagcatcaagagaattagtcaagtgtggtgtcacgtccctgtagtcccagctacttgggaggctgaggtggaagaatcacttgagctctggagatgaatgctacagcagagagccgtgattgtgccattgtgcttcagcctggacaacagcaagattctgtccaaaaaaaaaatagtttcaggttcaacatttatttaataattttatttagtaaatattaattcaacatctacgtgtcaggcatgctaccagcctctacaaactcaaaaatgaatacaaagtaattccatccacaagaaattctcaatctgtggactctctggtgtgccaacaactatattagttgcctgatatgtttgttcatttatttctcacaaccttaagaggtaaaatttcttgtccccagatgagaaaagttaattcagagatgttaaataaattgcccagggttacagagttactaaacagagcagtagattataatcccttttaggactcaaatacatctcatcagctgtcatcaagattaaaaaatagttcatcatttagatcaatattggataataatgttcattctattccatatcaggagacctaggttcaagtcctgctttggtgtttattgagtaacctgaagctattccgtttcttcatttgctaagtgggagtaggggcagctctgaggactgagatagctaactcttcttccctcccggagcaaattatatgtaccactgtgtatactacttgtcactttacattgtaatcagtatttgcccatttctcccagtatactttgagtgcttgaggaccagaatctttcttcatccttttgtgttcagcatttcctgcagtgcgtgacacacatttggtgttcagtaaaggctttctgcatggataagtgcttgagaagtgtcattagtgagctgaaatccttcaaaagagagttgtgagaaaacagagaaatacttccattaagaacaaaaatcaaatcattcaacaaggatgggaacatgaactattattataaaaggtctgtttaaaataattatttcaaaagccaggtgtaatggtgtgcacctgtagtcccagctacttgggaggctgagataggaggatcactagaggccaggagttcgaggctatagcgtgctatggttacaccagtgaatagccagtgcaccacctgtaatcccagctacttcggaggctgagacaggagaattgtttgaacccaggaagtggaggctgcagtgagcagatatcacgccactgcactccagcctgggtgagacagagcaagactgcgtctcaaaaaagtctaactcatcaagaaaaaatagatatctgtatgcattttttctattttgtcttaaaatatgagaaaaagcctcttattttaatatgaaattactctgggaaagagcttggtaaattggggtggaacggggaattaggcctcccaaaattgacctcctaatatttcaggggaggttattaagtaatttgatttgtgaagttatagtagactattgattctttcacactttacatttattatttccagtaagggatatagctaagatagttattgatgttgttgtctttggatttaccatttttattcagatatatttgtgaatgagattaggactcttaaagaagaaggagcatctgtattcatttaatctttgtatatcattggagaaatagccatttagttggctttttggcccaagcatctgaagccaagatgcagttacagatgtttatatatttgatattaaatttgtggcatttgataactttattaaataattcagcatctcttgactttgtattataggtgaggaaacttctgtcacatagtaggcttagaataaacctgtgtttctggtttgttacccactaaactgtcttattgatacaaatatcagtaattacaagtttaatttagaaatatggttgctcctaaactttcagactgggtagactaagcttcaccaagacacactgtgtgtgtctcttgatgaaatgagaaaggggaaatggtatttgctacattgtcgaccactcatttatcacttcactgaattagccagtcctaagaagcagctctgttgactctcctgtctccttgccctcagtccacctggaaggccgctgtggtggaaggatgtttgactgcagagatgtggcattcactgtgggcgaaggagaagaccacgacattccaattggaattgacaaagctctggagaaaatgcagcgggaagaacaatgtattttatatcttggaccaaggtaagcagcataccagtttggaacacagtgaagtcacctaaaggacagggatatgcttgctttttcattcagtgttttggcattttcagtgaagttaatccaacaacggaaacaattaaatcaggctgcagttagtcatcacgtaaattaatgggccttacaatatcatagaggtttgtgagcatgtttacttgattgatatatgttcagaacaggtcttacagccactttttgcttttgtgatgatttgttttgcctgcagtattgccaagacaggcagctatggacccctctcatatgtggttctcttaccccctcttcccttttctctaagttaggcaggaggcaggaaaacatgggggttgggagtcaaagaagcatttggatgatttactaccacttgaacttgtgccacttgttcttatacagagtccctgaaaaaaaaagcacattcttcctggatctaaaggcaacagagatgactcattttttgtctaagtttttcccatcataaggatatccattagcactttcaagcttagaaccatcagacactgacagtctctcttgtgaaaacatgtttagtagtatttttagatctaaaattataccatgaatccaactcttctgtcttgagaaacagacccctgagagaacccggggtgttttgattttgtctgcatgagagtgcatctgcttatctagtgtatcctccctccggctccatggatttcagacagtgggattctgagttactgagaatcttgatgacctgcacttgccagtaggtgatgttaagccttttgtggtgtcattttatagactctgtagtaatccaatggcagtttacctttgccccagcacttgatccataatcatatatatatatattttgagatggagtcttgctctgccacccaggctagagtgcagtggcatgatcttggctcactgcaacctctacctctgggttcaagcaattttcctgcctcagccattttatggttttttttaatatgtaaatatggttgatctagaatttacttttgtgcttacaatatgaggtaagaacttttttttttttttttttttggagacaagagtctcactctgtcacccagcctggagtgcagtggcacgatctcagcccactccaacctccacctccctggttcaagcaattctcctgcctcagcctcccaagtagctgggattacaggcgcctgccaccatgcccagctaatttttgtattttcagtagagacaggatttcgccatgttggccaggctggtctcgaactcctcacctcaggtgatcacctgcctcagcctcccaaagtgctgggattagggtgtgaaccactgcacccggccttaactcttattttctatgtggatagctgccatcctggccccgtttattttaaagttaactctccactgattcacattaccacctttgtcattttcaaatgtttttatatgcagacacatgactgtttctgaatgattcgttctgttccactgagctgtttgtccacatgctagtaaccaaactgtcttaatttcagtagttttaaagtttgtctttttctctggtaagacaagtgcctcttatttttttcttttttcccaaattttttcagccattcttgggcacttatttgttccaatgaactttaaaatgaacttctcaagagccccctctcaaatctccttgggatctttctagaaattgcattaaatgcagaaaataatttgggtagtaaattttgatcagtagtatgtaattatcttgagccccatttttttaaatacactttttccatctcctttggcaagagaactttggtaagctgtttactgaatttattaaaattcgtcctttctaaaatatagaccatctattcctaggcccttggactggacagtgccaatgagaaaggcttgtataggaggggtgaagcccagctgctcatgaacgagtttgagtcagccaagggtgactttgagaaagtgctggaagtaaacccccagaataaggctgcaagactgcagatctccatgtgccagaaaaaggccaaggagcacaacgagcgggaccgcaggatatacgccaacatgttcaagaagtttgcagagcaggatgccaaggtgtgtgcagagcctgatgactgcaggtggatcaagagcctgaccatgcccctttgattttaggggacaggaacagctaaacaacccaagaggctcaagagcagggaaaacgatttccaatctaatctgaggctgctttggatccaaaggtcgagtagggcagttccagtcaccaaaggcacacgtcatgagagatgagaaaactaaattcccttcagatgtattagattggttcaaaagtaattgtggtttttaccattactttcaatggcaaaaacagcaattttgcaccaactccccccgcccgccccccggaattctgaagttcctattccgaagttcctattctctagaaagtataggaacttcggttaccatatggccggccatcttaattaaggcgcgccggcccctgcccgagtcagagtctcactctgtcacccagactgtaatgcagtcgtgctaacatggctcactgcagccttgacttctctggatcaagtgatcctcccacctcagcctcctgagtagctaggacaagcacactaccacactgggctaattttttaaaattttttgtaaagatagggtttcactttgttgtctaggctggtctcgaacccctggcttcaagggttcctcctgctttggtctcccaaagtgctgggattatagacatgagccaccatgcccagcccttttgtcatttttgaaaaattgtaaacccatcaggatttttttttcaagtaagacttacgattttcccaggaaatgggatgttataggagccccatcatcacacctgttatcttgagggtcggcagccagcctgaaaaatgtggcaggagaggtctgtctgcagagagggcggcctccctacagcatagttggcattctggtcctgtccccagtctccaggaatgcccagacttcattatcccacagctgtcccatgtgtcaaccaaagaaaggtgaagctcccactctaactattggtagcacagccctgacctacccagctagatatcctagagaaaatcattgcaatacctcgtgtttgggacctgatacacagtctggaggagccaggaattatttcacttctttattctctaaccttcttgattgcttatttcttccttaggaagaggccaataaagcaatgggcaagaagacttcagaaggggtcactaatgaaaaaggaacagacagtcaagcaatggaagaagagaaacctgagggccacgtatgacgctgcgccacggagggaagagagtcctaatgaactcggccctcctcgctgggctcgcctccaactcaggactgaacagtgtttagtgtaaggtttgttacagtctctgtgattctggaagcaaatggcataccagtagcttcccaaatgaccacctgctgctgcgggggggtgggggtgggggacatgccaggaaacagcagagaaggccgctggtgtgaagagaccaggccagcagctcagtccagcccatttcagtttgtcacctttcagtgtccagcacagcatccctgtgaacctagggcccagctgctgtgggttctacatcggcactagggtcacactgcagaaaccgttgataaaacaaactcagtgatctctgctttcctattggtgggcatggcaggggcgggtgatgagatttgcttagcactgactgactggcctgctaagaacacaagcccacagccaggggctccctggtccacagctgggtctcaggccccttacctgccttccaagtcctttcgcagactcttgagtgtggctttctgtcctagccagcatgtcccacagactctgttgttcctccaacgcccgtcattagtgacagctttctctctgagtttctgtggtgtggagagtgggtagaagtaggtttatctttcccgctgtctgccccactcaaggacgatgttagcgctcagccatccctgcccagcacagctgctcagcctcatgctcccacccgcacccttgctgtgcagaacctaaggcttgctcccgtcccagactcgagtggacggacatccatgtccacccaggattgacgaaggaaggaacttcacactcctctatcatgcaaaaatagttttcaatttttactttttagcttgggttttgaaaggcaaactctaggaggcttaaaacgggcagttactcttaaggcgaggtagatacctcatcctgcaccgcagcctctgtattctggtgggagctgtaagggttgcgggcttccggtgtccatgcatcaagccaaagtctccaaaacttgggcattgaattagggcaaatcttaaatagttttggggttggattttagtcctacaggtgatgattgaacgtggctggcttctccacagcctaaggaacccccatggggacacacccaccacccaatgacccatgcacctctgcctggacagctcacttccagccctaaggaccactgtgctcaagatgtgtggctgtagtaagacagtctcatgtcaggcattaagtcaaggccttccttcacacctgcgggacagcgcttctgaggctccagttctgctctgctcgaagcaggagggatgggttgtgaaggagtctgggatgtgactgggaccctcgcatctcacactcggcactctgcctctttccatccatgtttgagagccctggtctcaccaggaagtgcagagcaggagccgactgtgtgtgtaatgctctagtgtttgtagttctcttcactagccttgcagcgccagggaggccgggctggaatttgagcctaacatgttaggtttttacgggaaagctctttttccagctgtctcccaagtgcgtattgtgggccaaggagactggtatgggaaggggggatggtcaaggttccaagccagaaatggaaggtgcccagcacccctggattttacatgttcttgtattcagtaatctcagacgatttacacggaaatcagaagatgtcttcaaatacccttagactggatagagtgatggggaagcagatgatgcattgaaattttggtttttaaaaaatcacttagatgctgggagtaatggtgacacacacccttggtcccgtgacctgggaggccctgtctcaaaacaaaacaaaaaatcatttagatgcattttgtgttccttaaataaattttttaaaaagtggtttttcattgttcatggctttttgtttttgttgtttgtttgatttttgaagtttacttttatctttgtctgctagagttttgtgtccagctgcccttggggaccagaaagggactttgaatctccttgaaatgaagatacagttgttagccagatgtgggtgttgagaactgaacccaagtcctctgaaaagcaggaagtgctcttaactgctgggctgcccctctcccacccctccattgtttgggtttgacttaaacacttgattttttttatgatttttttaaacagatttatttattgtatgtatgtgaatacgctgttgttgtcttcaggcacagcagaagaaggcattggatcccattacagatggttgtgagccaccatgtgggtgctgggaattgaactcaggccctctggaagatcagtcagtgctcttatccactgagccatctctccagcccctttttatgattttattttatgtgtatgcacacttttttgtgatgctttattgagttcctatggaacccttctaaccccctacctcaggtaggggagaaagaaggttagaggggggaagggggcatagatcttttcagactgcttcttgctgattaggggcatcgagttccttgggtcaacttcaaacttcattgtcagaatatccaatctactcgtctctttgctcacagccttgtgacaagccacagcagcaacagacacagtagcctcctctgcagagcgcactcccacctgatagcccctctcagagctcggcatttataccctatgagaagttcccagaatcccagagttcacagaaactgtctgcagctggcaaa


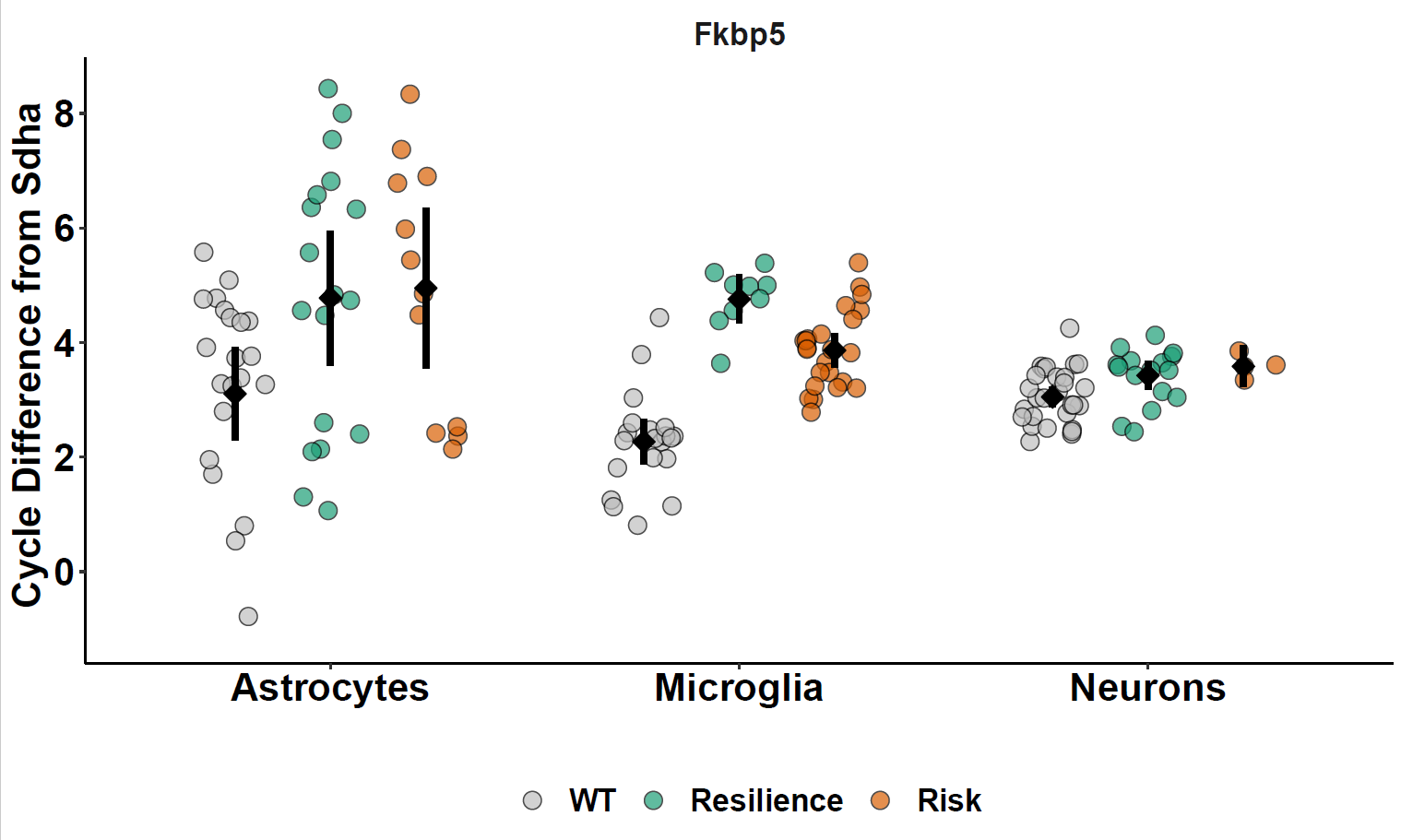


**Fig. A2.** *Sdha*-normalized mRNA expression levels of *FKBP5* of primary murine astrocytes, microglia and neurons. Data from cells of the risk (A/T) allele carrying strain are visualized in orange; cells of the resilience (C/G) allele carrying strain are visualized in green and wild type cells are shown in grey. Individual data points are visualized alongside with their mean ± 95% confidence interval. High values in the PCR cycles needed to reach the set threshold represent low amounts of the targeted mRNA and hence a low expression of the gene while low cycle numbers indicate higher expression.


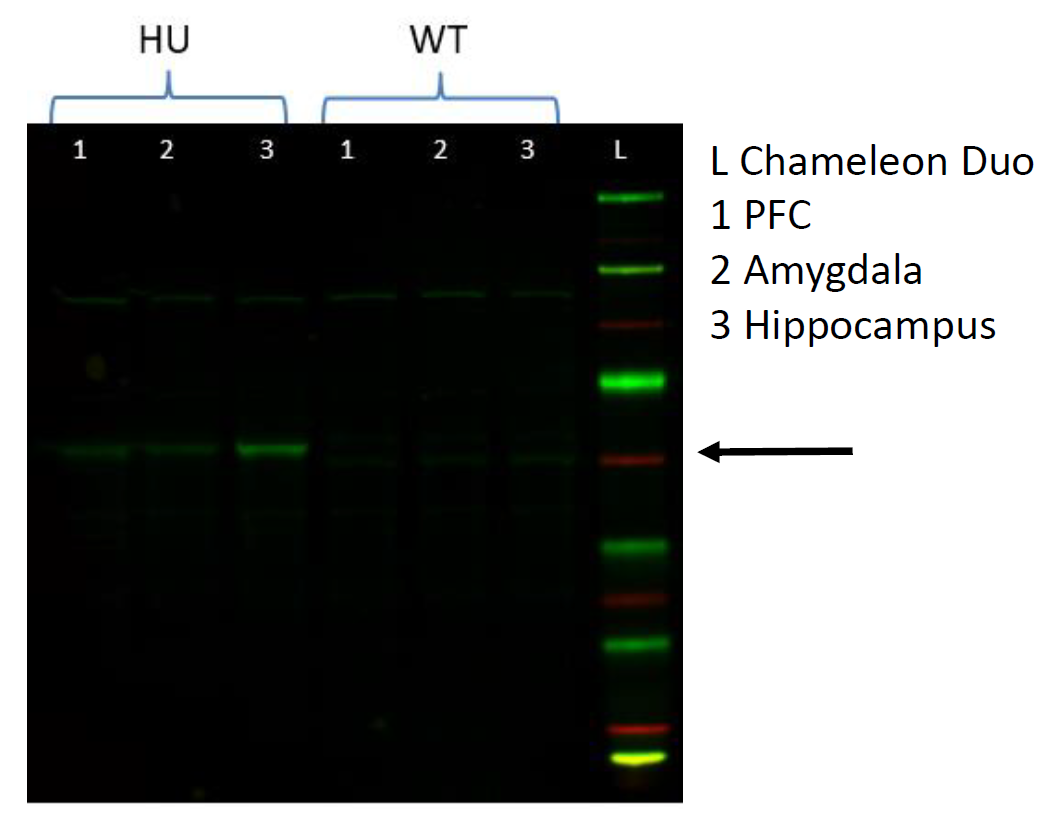


**Fig. A3.** Representative western blot of SDS-electrophoresis separated proteins from prefrontal cortex (1), amygdala (2) and hippocampus (3) homogenate of a humanized mouse (left side) and a wild type mouse (right side) stimulated with 0.1mg/kg subcutaneous dexamethasone for 24 hours. FKBP51 is labeled in green and indicated with an arrow at a height of 51 Dalton. Chameleon Duo Ladder was used as protein length reference and input material was normalized to 30μg based on BCA-assay results, confirmed by expression of β-actin as housekeeper and total protein staining on the membrane. In addition to a band at 51 Dalton, a band at around 102 Dalton was detected, which could represent a FKBP51 dimer.

**Protocol Western Blot**

Humanized mice and wild type mice were subcutaneously injected with 0.1mg/kg dexamethasone in the morning and sacrificed 24 hours later. Prefrontal cortex, amygdala and hippocampus were dissected and protein content determination using BCA-assay (Pierce™ BCA Protein Assay Kit, Thermo Scientific™, #23225) following the manufacturers’ instructions was carried out. The remaining sample was digested with DNase I (Qiagen, #79254). Laemmli buffer (BIO-RAD, Germany, # 1610747) supplemented with 10% ß-mercapto-ethanol (Sigma-Aldrich, #M3148) and heating of the sample at 95°C was used for linearization. Next, samples or a molecular weight ladder (Chameleon Duo ladder, LI-COR, # C50731-03) were electrophoretically separated (NuPAGE™ 4-12% Bis-Tris Gel, Thermo Fisher, # NP0321BOX, 120V, 105 minutes). After dry blotting onto a nitrocellulose membrane (iBlot™ Dry Blotting System, Thermo Fisher Scientific; iBlot™ Transfer Stack nitrocellulose mini, Thermo Fisher Scientific, #IB301002), total protein staining was performed following the manufacturers’ instructions (REVERT, LI-COR, # 926-11016) and fluorescence was measured on an Odyssey instrument (LI-COR). Following removal of total protein staining, the blot was blocked over night at 4°C (Rockland, USA, #MB-070) and incubated with the first antibody to detect FKBP51 (rabbit monoclonal antibody targeting FKBP5 (D5G2), Cell Signaling, #12210, 1:750 dilution in blocking buffer) for 5 days at 4°C while gently shaking. After washing (Tris-Buffered Saline with Tween, Cell Signaling, USA, #9997; Tris-Buffered Saline, BIO-RAD, #1706435), the second antibody for fluorescent detection (IRDye 800CW, Goat anti-Rabbit IgG, LI-COR, Germany, # 926-32211, 1:15000 dilution in blocking buffer) was incubated for 2 hours at room temperature. After washing, the green fluorescence of the secondary antibody was detected at 800 nm with a fixed intensity of 2.5. Next, the membrane was incubated with anti-β-actin (abcam, USA, ab8227, 1:5000 diluted in blocking buffer) over night at 4°C, washed, labelled with IRDye 680RD (Goat anti-Rabbit IgG, LI-COR, # 925-68071, 1:15000 dilution in blocking buffer), washed and the red fluorescence was detected at 700nm with an intensity setting of 2.
