# Supplementary figures and images for: FKBP5 polymorphisms induce differential glucocorticoid responsiveness in primary CNS cells – First insights from novel humanized mice
Source: Eur J Neurosci. 2020 Oct 27;53(2):402–15. doi: 10.1111/ejn.14999 (PMC7894319; doi:10.1111/ejn.14999)

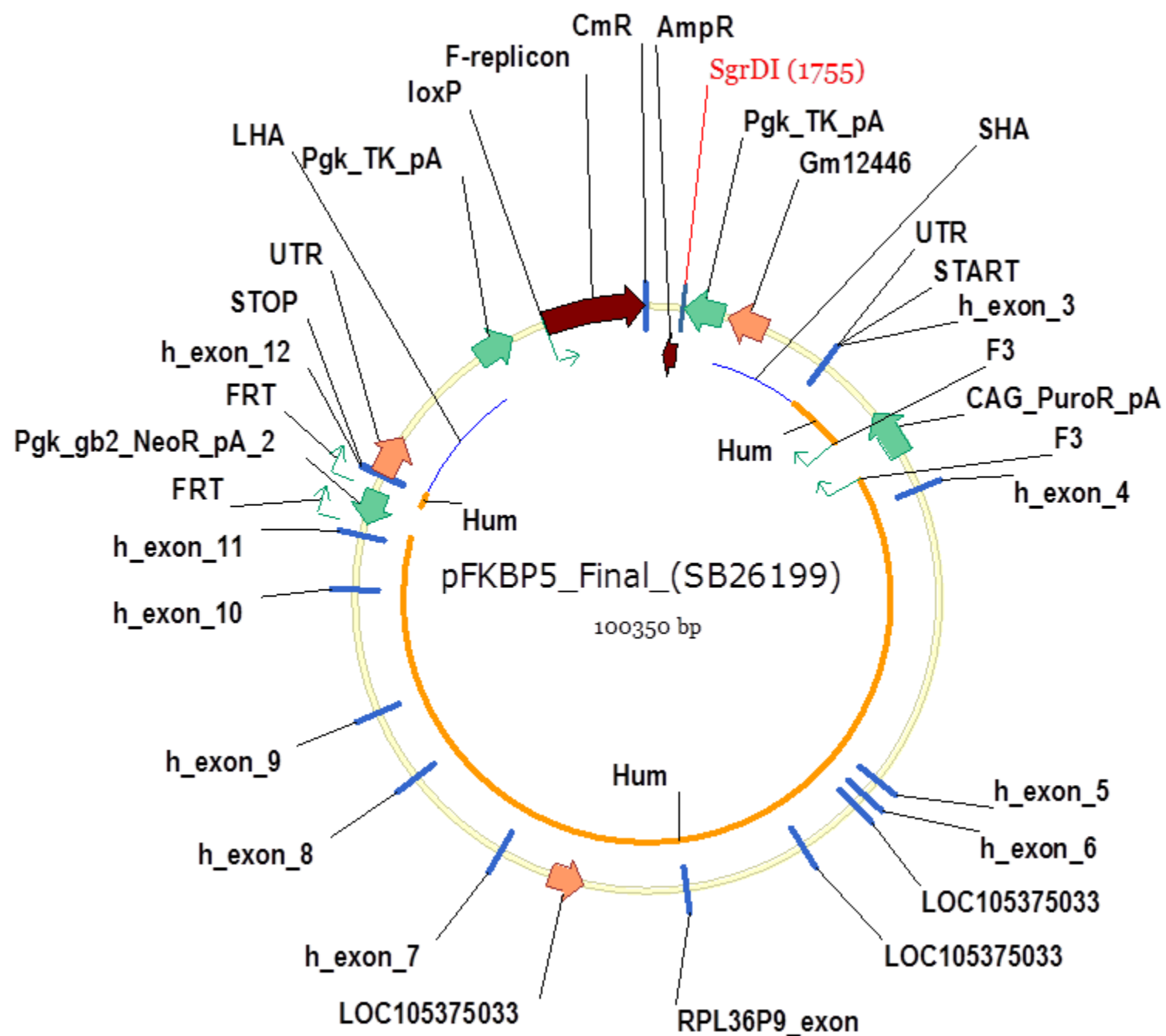

Supplement: Supplementary file 2 — Supplementary Material [file EJN-53-402-s002.pdf]

# Fkbp5

Cycle Difference from Sdha

Astrocytes

Microglia

Neurons

○ WT    ● Resilience    ● Risk

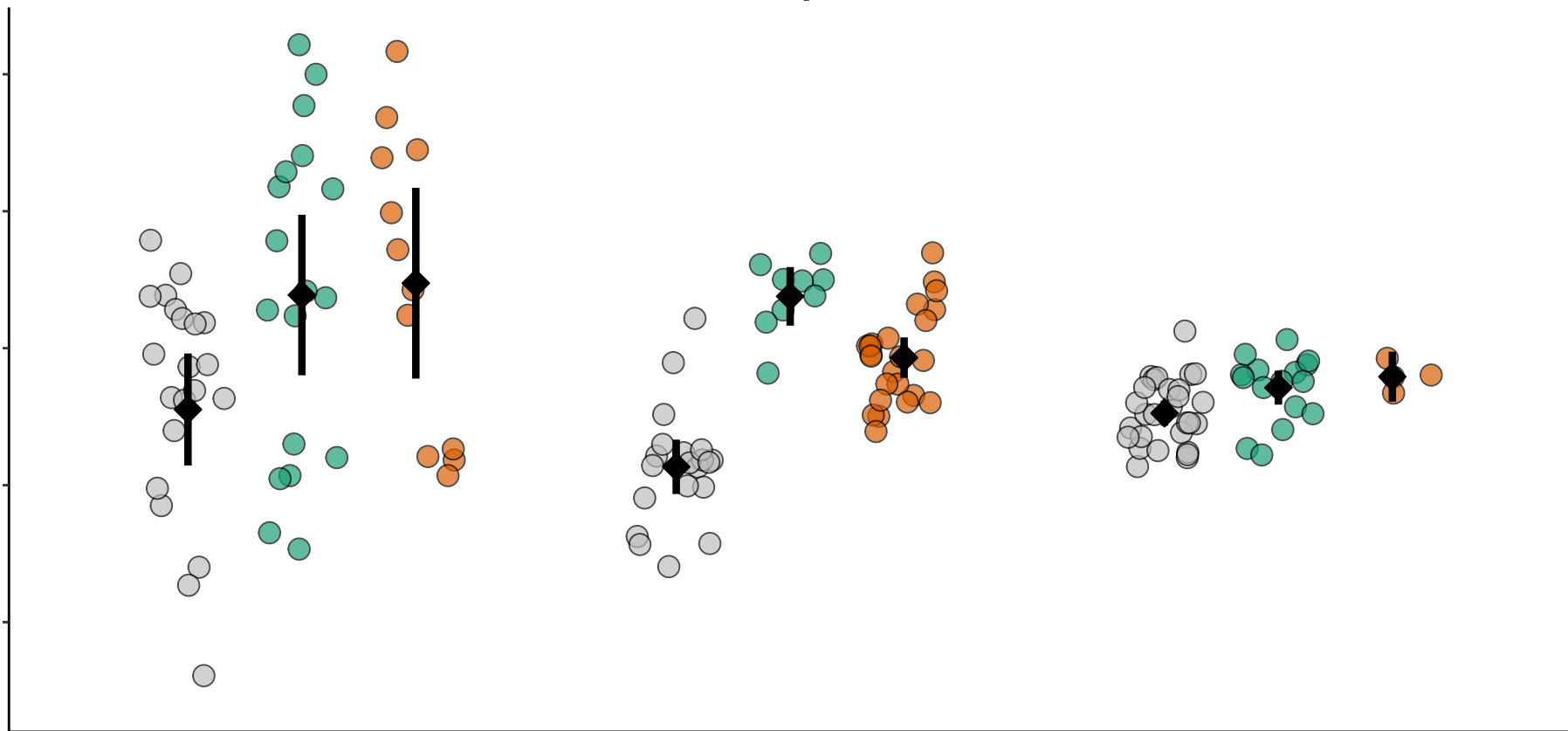

Supplement: Supplementary file 3 — Supplementary Material [file EJN-53-402-s003.pdf]

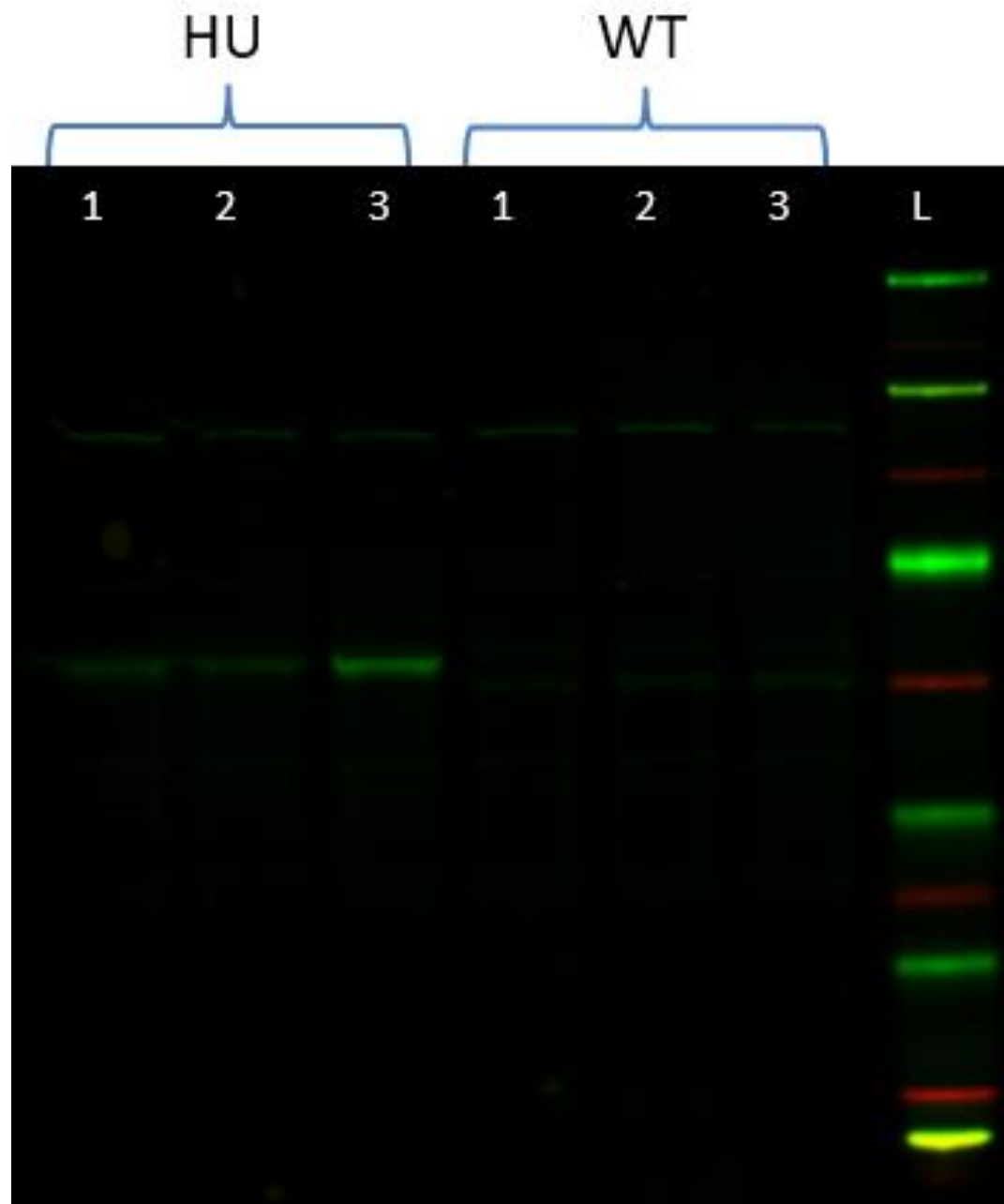

L Chameleon Duo

1 PFC

2 Amygdala

3 Hippocampus

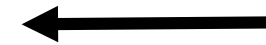

Supplement: Supplementary file 4 — Supplementary Material [file EJN-53-402-s004.pdf]
